# scSelector: A Flexible Single-Cell Data Analysis Assistant for Biomedical Researchers

**DOI:** 10.3390/genes17010002

**Published:** 2025-12-19

**Authors:** Xiang Gao, Peiqi Wu, Jiani Yu, Xueying Zhu, Shengyao Zhang, Hongxiang Shao, Dan Lu, Xiaojing Hou, Yunqing Liu

**Affiliations:** School of Computer Science, Luoyang Institute of Science and Technology, Luoyang 471000, China; litgx@lit.edu.cn (X.G.); peiqiwulit@163.com (P.W.); 18847003718@139.com (J.Y.); 13656998001@163.com (X.Z.); m17886659527@163.com (S.Z.); 200904601573@lit.edu.cn (H.S.); ld197832@163.com (D.L.); alisha@163.com (X.H.)

**Keywords:** interactive cell selection, cellular heterogeneity resolution, LLM-assisted interpretation, outlier cell analysis

## Abstract

**Background**: Standard single-cell RNA sequencing (scRNA-seq) analysis workflows face significant limitations, particularly the rigidity of clustering-dependent methods that can obscure subtle cellular heterogeneity and the potential loss of biologically meaningful cells during stringent quality control (QC) filtering. This study aims to develop scSelector (v1.0), an interactive software toolkit designed to empower researchers to flexibly select and analyze cell populations directly from low-dimensional embeddings, guided by their expert biological knowledge. **Methods**: scSelector was developed using Python, relying on core dependencies such as Scanpy (v1.9.0), Matplotlib (v3.4.0), and NumPy (v1.20.0). It integrates an intuitive lasso selection tool with backend analytical modules for differential expression and functional enrichment analysis. Furthermore, it incorporates Large Language Model (LLM) assistance via API integration (DeepSeek/Gemini) to provide automated, contextually informed cell-type and state prediction reports. **Results**: Validation across multiple public datasets demonstrated that scSelector effectively resolves functional heterogeneity within broader cell types, such as identifying distinct alpha-cell subpopulations with unique remodeling capabilities in pancreatic tissue. It successfully characterized rare populations, including platelets in PBMCs and extremely low-abundance endothelial cells in liver tissue (as few as 53 cells). Additionally, scSelector revealed that cells discarded by standard QC can represent biologically functional subpopulations, and it accurately dissected the states of outlier cells, such as proliferative NK cells. **Conclusions:** scSelector provides a flexible, researcher-centric platform that moves beyond the constraints of automated pipelines. By combining interactive selection with AI-assisted interpretation, it enhances the precision of scRNA-seq analysis and facilitates the discovery of novel cell types and complex cellular behaviors.

## 1. Introduction

Single-cell RNA sequencing (scRNA-seq) technology enables high-resolution analysis of individual cell transcriptomes, overcoming the limitations of traditional bulk sequencing methods and providing novel insights into complex biological systems. It has become a key tool across multiple fields for deciphering cell identity and function. Important aspects of scRNA-seq data analysis include (1) distinguishing and filtering out outlier cells caused by technical artifacts while preserving those reflecting genuine cellular physiological states; (2) accurately determining cell types and ensuring consistency with clustering results; and (3) identifying the functions of specific cell populations through differential expression analysis, enrichment analysis, and other methods. A variety of tools support scRNA-seq data analysis, ranging from detailed interpretation pipelines, like Seurat [1] and Scanpy [2], to specialized tools focusing on cell type annotation, such as SingleR [3] and CellAssign [4]. More recently, methods applying large models to single-cell data analysis have emerged, including scGPT [5], scBERT [6], Geneformer [7], and GPT4 for cell type annotation [8]. Collectively, these tools provide effective support for navigating the complexities of scRNA-seq data analysis.

However, effectively translating these complex computational workflows into accessible insights remains a challenge for many biomedical researchers. While established pipelines offer powerful capabilities, they often require significant programming expertise or involve manual parameter tuning that can be daunting for wet-lab users. Firstly, the cell filtering step during preprocessing poses a risk of losing valuable information. As detailed by Ilicic et al. [9], distinguishing low-quality cells from biologically significant outliers is challenging, and standard automated metrics may inadvertently filter out rare cell types or unique biological states [10]. Existing tools often lack the intuitive visualization capabilities to allow users to easily inspect and reclaim these populations. Reliance on rigid clustering parameters for cell type identification introduces significant limitations. The selection of cluster numbers is highly subjective and can lead to vastly different outcomes, presenting a major challenge in unsupervised clustering [11]. This subjectivity directly impacts downstream differential expression analysis and cell type interpretation, affecting the reliability of the conclusions [12]. Moreover, the poor controllability over clustering adjustments, often dependent on single parameters, restricts users from leveraging their expertise to define cell type boundaries by merging or splitting clusters.

Furthermore, in the realms of cell subpopulation identification and developmental trajectory analysis, the intricate relationships between cells demand expert interpretation from researchers. Tritschler et al. [13] meticulously analyzed the concepts and limitations of inferring developmental trajectories using single-cell genomics, emphasizing that algorithms cannot entirely replace the specialized knowledge and judgment of researchers. Likewise, a comparison of various single-cell trajectory inference methods by Saelens et al. [14] revealed that different algorithms can yield conflicting results when processing complex cellular relationships, highlighting the necessity of selecting appropriate methods based on specific data characteristics. These studies underscore that professional intervention by researchers remains essential for accurately navigating and analyzing complex cellular relationships.

To address these challenges, this study introduces scSelector (v1.0), an interactive single-cell data analysis toolkit designed to provide an intuitive, GUI-based platform that democratizes advanced analysis for wet-lab researchers. By automating complex interpretation tasks through large language model (LLM) integration, scSelector enables users to select and analyze cell populations flexibly and in real-time, guided by their expert knowledge. Our approach integrates a visual interface with rich analytical functionalities, enabling researchers to (1) retain and analyze unique cell populations potentially discarded by conventional filtering methods; (2) flexibly define cell groups without being constrained by predetermined cluster numbers; (3) intuitively partition relevant cell populations based on their own experience; and (4) leverage the automated power of large language models (LLMs) to predict the cell types of user-selected populations. The interactive methodology proposed here significantly enhances the flexibility and precision of data analysis, providing a effective tool for discovering novel cell types and investigating complex cellular relationships.

## 2. Materials and Methods

**Figure 1:**
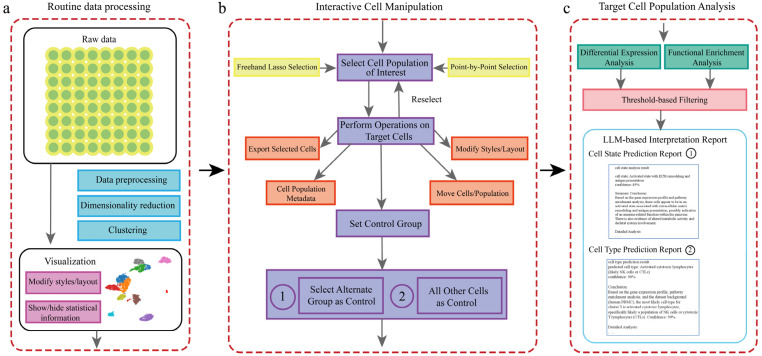
Overview of the scSelector interactive analysis workflow and core functionalities. (**a**) The routine data processing module. This pipeline accepts raw or preprocessed single-cell data and performs standard steps, including data preprocessing (e.g., QC, normalization), dimensionality reduction (e.g., PCA, UMAP), and clustering (e.g., Leiden algorithm), to generate a 2D visualization. (**b**) The interactive cell manipulation module. Users can select any cell population of interest directly on the visualization plot using either a freehand lasso or point-by-point selection. Once selected, various operations can be performed on the target cells, including exporting, modifying visual styles, or repositioning. A key feature is the ability to set a control group for comparative analysis. As illustrated, there are two distinct options for setting the control group: (1) selecting another specific cell population as the control or (2) using all other cells in the dataset as the control. (**c**) The target cell population analysis module. For a selected population and its control, scSelector performs differential expression and functional enrichment analyses. These results are then used as inputs for the integrated large language model (LLM) to generate a comprehensive interpretation report. The type of report generated corresponds to the control group selected in step (**b**). Specifically, comparing the target group to an alternate group (b-1) yields a Cell State Prediction Report (c-1) focused on functional differences, whereas comparing it to all other cells (b-2) is used to generate a Cell Type Prediction Report (c-2) for identity prediction.

To facilitate efficient interactive analysis of single-cell data, we have developed the scSelector system. Its complete analytical workflow is illustrated in Figure 1, encompassing the entire process from raw data processing to the generation of interpretive reports.

### 2.1. Software Development

#### Main Framework Development

scSelector integrates dimensionality reduction, clustering, differential expression analysis, functional enrichment analysis, and interactive visualization. Users can also import analysis results, such as clustering assignments and UMAP coordinates, generated by other software into scSelector for further analysis. Developed using Python (v3.9), scSelector relies on core dependencies, including Scanpy for single-cell data processing, along with Matplotlib and NumPy, for plotting and mathematical computations. All packages used by scSelector are shown in Table 1.

scSelector supports the input of preprocessed data, allowing users to bypass filtering [21] and normalization steps on the raw data. This approach preserves more complete cellular information and facilitates the reproduction of previous analysis results by other tools. Alternatively, when users choose scSelector for data preprocessing, the workflow typically includes quality control (QC) metrics, such as the proportion of mitochondrial genes, followed by filtering cells and genes based on user-defined thresholds. Subsequently, library size normalization and log transformation of the expression data are performed. Optionally, highly variable genes (HVGs) can be identified for downstream analysis. Dimensionality reduction is carried out using Principal Component Analysis (PCA) to extract key features, typically retaining the top 50 principal components by default. Based on the PCA results, a K-Nearest Neighbors (KNN) graph is constructed (default K = 15), and the UMAP algorithm is applied for non-linear dimensionality reduction to generate a two-dimensional visualization. Finally, the Leiden algorithm is employed to perform clustering analysis on the cells, identifying populations with similar transcriptional profiles.

### 2.2. Differential Expression Analysis

Identification of differentially expressed genes (DEGs) is performed using the Mann–Whitney U test, which compares the gene expression differences between the target cell population and all other cells. For each gene, the log_2_(Fold Change) (log_2_FC) and the *p*-value are calculated. Subsequently, the *p*-values are adjusted for multiple testing using the Benjamini–Hochberg method to obtain adjusted *p*-values (adj.*p*). The criteria for filtering DEGs are set to an absolute log_2_FC greater than 1 (|log_2_FC| > 1) and an adjusted *p*-value less than 0.05 (adj.*p* < 0.05). By default, scSelector displays the top 50 most significant DEGs, ranked according to the absolute value of their log_2_FC.

### 2.3. Enrichment Analysis

Functional enrichment analysis based on the identified differentially expressed genes is implemented using the GSEApy [17] package. Users can specify the gene sets for this analysis in two ways: either by providing the path to a local GMT-format pathway gene set file along with the pathway type via command-line arguments when launching scSelector or using specific command-line parameters to utilize online public gene set libraries available through the Enrichr platform. By default, scSelector displays the top 30 most significant pathways, ranked by statistical significance. The available gene sets for enrichment analysis include Gene Ontology (GO) [22], which encompasses a biological process (BP), molecular function (MF), and cellular component (CC), as well as the Kyoto Encyclopedia of Genes and Genomes (KEGG) [23].

### 2.4. Using Large Language Models for Result Interpretation

scSelector supports three primary methods for cell type annotation: directly reading annotated h5ad files, importing external cell type annotation files, and utilizing prediction models for automated annotation. The prediction functionality integrates the DeepSeek API and Gemini API to perform cell type prediction based on differentially expressed genes, functional enrichment results, and clustering information. The prediction output primarily includes the predicted cell type or cell physiological state and confidence (confidence score) in a structured format. Additionally, the complete textual response generated by the large language model, which typically contains its detailed reasoning process, is returned to assist researchers in cell type identification.

### 2.5. Datasets

To validate the performance and accuracy of scSelector, multiple publicly available single-cell RNA sequencing datasets were utilized. First, the human Peripheral Blood Mononuclear Cell (PBMC) dataset was used; human peripheral blood datasets are commonly employed for validating single-cell algorithms due to the relatively distinct differences between cell types. The PBMC dataset used in this study originates from the work of Ding, J. et al. [24]. Second, a liver dataset was included. As the body’s largest gland and parenchymal organ, the liver is responsible for metabolism, is primarily composed of hepatocytes, cholangiocytes, etc., and possesses strong regenerative capabilities. The liver dataset used in this study is from the work of Muraro MJ et al. [25]. Finally, a pancreas dataset was employed. The pancreas, an important endocrine and exocrine organ, is mainly composed of alpha cells, beta cells, and acinar cells, playing important roles in blood sugar regulation and digestion. The pancreas dataset used here comes from the work of Camp JG et al. [26]. By testing these real-world datasets, representing diverse tissue origins and cellular compositions, the functional performance and usability of scSelector across various application scenarios can be thoroughly evaluated.

## 3. Results

### 3.1. scSelector-Assisted Annotation of User-Specified Cell Clusters

In current single-cell data analysis workflows, cell clustering/cell type identification and UMAP visualization coordinate calculation exist as separate computational steps, often leading to inconsistencies between clustering results and UMAP visual distributions. This decoupled process may result in situations where certain cell clusters may not be effectively identified in UMAP plots. scSelector addresses this challenge by implementing a lasso selection tool that enables users to freely select cell clusters of interest on UMAP projections, thereby facilitating specific analysis of arbitrarily defined cell populations.

#### 3.1.1. Identification and Validation of the Platelet Cell Cluster

In some single-cell datasets, the number of certain special cells may be small. Analyzing and identifying these cells is significant; for example, platelet cells in the PBMC dataset. To evaluate the application of scSelector in cell type identification, we first conducted an analysis targeting a putative platelet cell cluster. Using the interactive lasso selection tool provided by scSelector, the target cell population was selected. Subsequently, the molecular expression profile of this cell cluster was analyzed using the built-in differentially expressed genes (DEGs) analysis module. The results showed that this population significantly expressed known key platelet marker genes, including *PF4* (Platelet Factor 4), *PPBP* (Pro-Platelet Basic Protein), *GP9* (Glycoprotein IX), *GNG11* (G Protein Subunit Gamma 11), and *TREML1* (Triggering Receptor Expressed on Myeloid Cells Like 1) [27].

Further functional enrichment analysis of the differentially expressed genes revealed significant enrichment in Gene Ontology (GO) terms closely related to platelet biological functions, such as biological processes and cellular components, including platelet alpha granule (GOCC_PLATELET_ALPHA_GRANULE), platelet activation (GOBP_PLATELET_ACTIVATION), hemostasis (GOBP_HEMOSTASIS) [28], and secretory vesicle (GOCC_SECRETORY_VESICLE).

**Figure 2:**
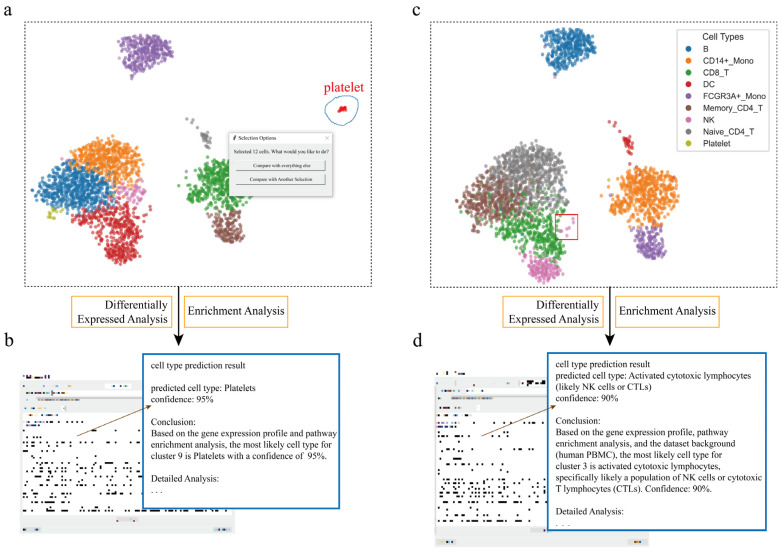
Interactive cell type annotation of distinct populations in PBMCs using scSelector. (**a**) The interactive lasso tool is used on a UMAP plot of the PBMC dataset to select a putative platelet cluster (blue circle). (**b**) The AI module output for the selection in (**a**), predicting the cluster as “Platelets” (95% confidence) based on its molecular profile. (**c**) A distinct subcluster of NK cells (red box), separate from the main population (colors in legend), is selected for analysis. (**d**) The corresponding AI output predicts the selection in (**c**) as “Activated cytotoxic lymphocytes (likely NK cells or CTLs)” (90% confidence), revealing both cell lineage and functional state.

Large language models (LLMs) were utilized to assist in interpreting those results. After providing necessary contextual information (such as dataset background, list of differentially expressed genes, pathway enrichment results) and API credentials, the model not only correctly identified the platelet identity of this cell population but also provided a detailed interpretation of the identification conclusion based on key molecular evidence, such as the highly expressed *PF4* and *PPBP* genes and significantly enriched pathways, like platelet activation and alpha granules (Figure 2a,b).

#### 3.1.2. Assist in Identifying the NK Cell Cluster in a Highly Proliferative State

Cells in specific physiological states, such as proliferation, often exhibit distinct distribution patterns in UMAP plots, appearing as outliers relative to their quiescent counterparts and thus posing a challenge for accurate cell type identification. To evaluate scSelector’s ability to resolve this, we targeted an outlier cluster of Natural Killer (NK) cells. Using the interactive lasso tool, we first delineated this population and then employed the manual cell repositioning feature to visually separate it from overlapping data points for clearer analysis. Subsequent differentially expressed gene (DEG) analysis revealed high expression of genes closely associated with cell proliferation, including *MKI67*, *PCNA*, *TYMS*, and *TK1* [29]. This was corroborated by functional enrichment analysis, which showed significant enrichment in pathways, such as the “mitotic cell cycle” and “DNA replication”, strongly indicating that the cells were in a highly proliferative state.

We then submitted this list of DEGs and enriched pathways to scSelector’s prediction module. The AI model identified the population as “activated NK cells” with 90% confidence. Notably, the detailed reasoning returned by the model demonstrated its ability to weigh two sets of molecular evidence simultaneously. It cited genes like MYBL2, KIFC1, MCM3, and *TK1* as clear evidence for the highly proliferative state, while also identifying lineage-specific markers such as *NKG7*, *CST7*, *GZMH*, and *IFNG* as strong evidence for their NK cell identity [30]. This result is notable as it demonstrates the AI’s capacity to integrate complex molecular signatures. While the strong proliferation signal could confound simpler annotation methods that rely on a few canonical resting-state markers, scSelector’s AI-assisted interpretation effectively balanced the functional state information with lineage-specific markers to arrive at a specific and contextually informed identification (Figure 2c,d).

#### 3.1.3. Identification of Rare Endothelial Cell Clusters in Pancreatic Datasets

To further evaluate the performance of scSelector when processing rare cell populations, we conducted an analysis of a low-abundance cell cluster within the pancreatic single-cell sequencing dataset. After precisely isolating this population using the interactive lasso selection tool, its molecular features were characterized via the built-in differential gene analysis module. The results clearly showed that this cell population specifically overexpressed a series of classic endothelial cell markers, including the key VEGF receptors *KDR* (VEGFR2) and *FLT1* (VEGFR1), as well as genes central to endothelial adhesion and structure, like *CDH5* (VE-cadherin) and *PECAM1* (CD31) [31]. Functional enrichment analysis of these DEGs also pointed strongly to endothelial cell biology, with significantly enriched pathways including “Vasculature Development” (GOBP_VASCULATURE_DEVELOPMENT), “Blood Vessel Morphogenesis” (GOBP_BLOOD_VESSEL_MORPHOGENESIS), and “Endothelial Cell Migration” (GOBP_ENDOTHELIAL_CELL_MIGRATION).

**Figure 3:**
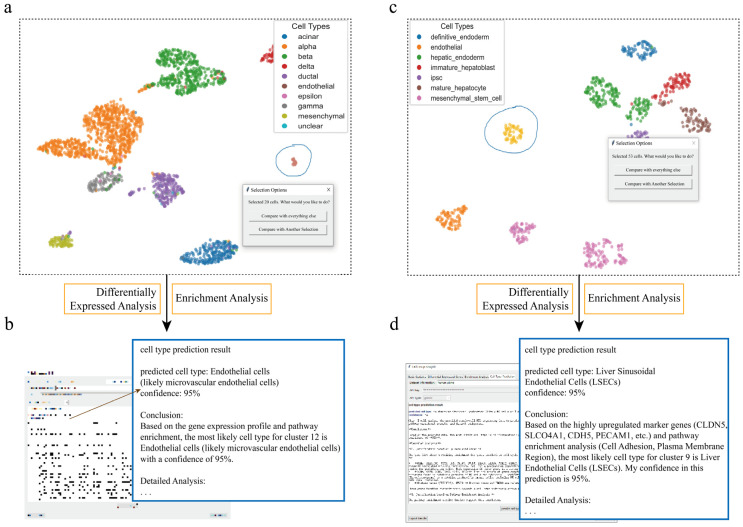
scSelector accurately identifies rare endothelial cell populations across tissue datasets. (**a**) UMAP visualization of a human pancreas dataset where the interactive lasso tool is used to select a low-abundance cell population (circled). (**b**) The corresponding AI analysis output for the cells selected in (**a**). Based on its molecular signature, the model accurately predicts the cluster as “Endothelial cells” with 95% confidence. (**c**) A human liver dataset where an extremely low-abundance cell cluster is selected (circled). (**d**) The corresponding AI analysis output for the cells selected in (**c**). Despite the low cell number, the AI module predicts the population as “Liver Sinusoidal Endothelial Cells (LSECs)” with 95% confidence, demonstrating the tool’s high sensitivity.

Leveraging scSelector’s integrated large language model for assisted interpretation, we provided these key molecular features (such as the DEGs *KDR*, *CDH5*, *PECAM1*, and the enriched vasculature development pathways) to the AI. The model accurately identified this cell population as “Endothelial cells” with 95% confidence. Notably, this AI prediction was matched with the original annotation for this cluster in the dataset. This finding provides support for the accuracy and reliability of scSelector in utilizing multi-dimensional molecular evidence for cell type prediction. Particularly for rare cell populations, this AI-driven confirmation, verified against ground-truth annotation, significantly increases confidence in the identification and demonstrates scSelector’s effectiveness in resolving rare, low-abundance cell types (Figure 3a,b).

#### 3.1.4. Analysis and Prediction of Extremely Low-Abundance Endothelial Cell Clusters in Liver Datasets

Finally, we evaluated scSelector’s analytical and predictive capabilities when processing extremely small cell populations. In a liver single-cell RNA sequencing dataset, we used the interactive lasso tool to isolate a minute cell cluster containing only 53 cells. Despite the exceptionally limited cell numbers, the built-in differential gene analysis module (by comparing this cluster against all other cells) identified its unique molecular signature, detecting elevated expression of multiple important endothelial cell markers including *CLDN5* (Claudin 5), *PECAM1* (CD31), *MMRN1*/*MMRN2* (Multimerin 1/2), *FLT1* (VEGFR1), and *S1PR1* (Sphingosine-1-Phosphate Receptor 1) [32]. Functional enrichment analysis yielded highly consistent results, with significantly enriched pathways concentrated in endothelial-related biological processes such as GO-BP: VASCULATURE DEVELOPMENT, GO-BP: BLOOD VESSEL MORPHOGENESIS, and GO-BP: ENDOTHELIUM DEVELOPMENT. The model accurately predicted this cell cluster as endothelial cells, which matched the true biological classification.

After completing the differential and enrichment analyses, the “Cell Type Prediction” function was activated, automatically submitting the calculated molecular feature information to a large language model (DeepSeek/Gemini). Based on this information on differential genes and enriched pathways, the model accurately predicted a cluster of only 53 cells as endothelial cells. Notably, this prediction matched the original annotation of the cell cluster in the dataset’s file, once again verifying the high accuracy and reliability of the AI prediction function, even when dealing with extremely small cell populations. The inference process returned by the model also cited key marker genes and pathways as the basis for its judgment.

This analysis of a small endothelial cell cluster in a liver dataset not only demonstrates the high sensitivity of the scSelector core analysis module in low-cell-count scenarios but also highlights a key advantage of its integrated AI prediction function in handling rare or difficult-to-define cell populations. By combining precise interactive selection, sensitive molecular feature analysis, and validated AI-powered interpretation, scSelector provides researchers with an effective toolset to effectively identify, annotate, and understand various cell populations from complex single-cell data, including those rare but potentially biologically significant cell subtypes (Figure 3c,d).

### 3.2. scSelector-Assisted Discovery of Biological Functions in Outlier Cells

In single-cell data analysis, outlier cells frequently emerge and can be annotated into distinct clusters through cell typing tools; despite their typically low abundance, these outliers often hold important biological significance, yet traditional methods fail to effectively compare functional disparities between clusters, leading to the drowning out of outlier-specific signals by dominant cell populations, while researcher-guided analytical approaches enable precise identification of these outlier cells’ functional characteristics.

#### 3.2.1. Assisted Discovery of Activated Cells with Extracellular Matrix Remodeling Function Under Mixed Conditions

A significant challenge in single-cell analysis is deconvoluting the functions of specific cell types under complex or “mixed” conditions, where target clusters may be contaminated by other cells. We leveraged scSelector to address this challenge within the pancreatic dataset.

We observed that alpha cells were divided into two separate subclusters, one of which was heavily admixed with nearly 50% of other cell types, posing a significant challenge for accurate downstream analysis. To overcome this, we employed scSelector’s interactive cell-dragging tool to manually remove the contaminating cells, thereby isolating a pure alpha cell subcluster for functional characterization. Analysis of this purified population revealed high expression of genes such as *GEMIN5*, *ARHGAP20*, and *CNGA4*. Functional enrichment analysis showed significant enrichment in pathways related to extracellular vesicle organization, cell adhesion, and collagen-containing extracellular matrix [33]. Based on this profile, the AI analysis precisely characterized their state as an “activated secretory state with extracellular matrix remodeling capacity” with 85% confidence, revealing how these cells may actively remodel their microenvironment.

In a similar vein, scSelector enabled the discovery of unexpected functions in other cell types within the same dataset. We identified a subcluster of pancreatic ductal cells that, in addition to exhibiting extracellular matrix remodeling capabilities, was also closely associated with immune activity, as evidenced by high expression of *ITGB2*, *HLA-DQA1*, and *CD84* [34]. This finding is particularly noteworthy, as ductal cells do not typically participate in immune activities under normal physiological conditions. The analysis from scSelector, therefore, generates important new hypotheses. This subcluster may either represent a pathological state associated with an immune response or it could be a misidentified immune cell type that warrants further investigation. This demonstrates scSelector’s capability to generate novel, testable biological hypotheses from complex data.

**Figure 4:**
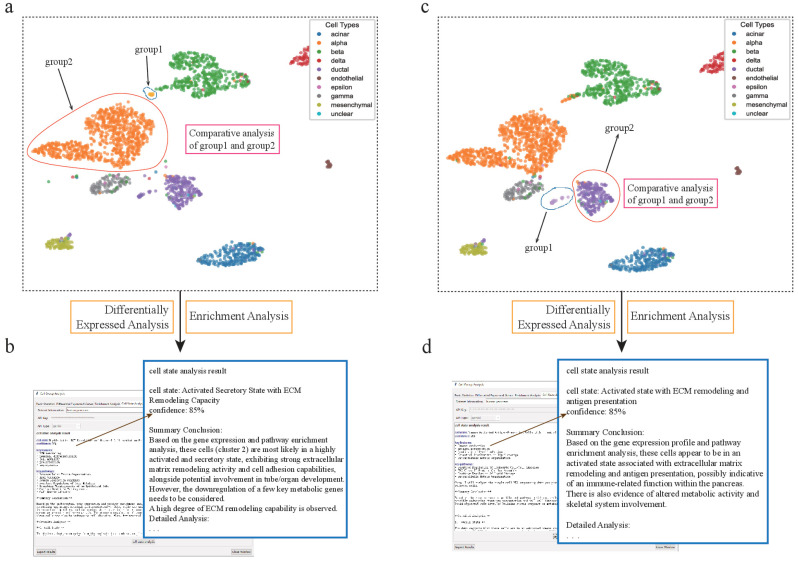
scSelector reveals functional heterogeneity in pancreatic alpha and ductal subpopulations. (**a**) UMAP visualization of the human pancreas dataset showing the selection of two distinct alpha cell subpopulations for comparative analysis: a major cluster (group2) and a smaller, distinct subcluster (group1). (**b**) The AI analysis output for the alpha subcluster (group1), predicting its functional state as an “Activated Secretory State with ECM Remodeling Capacity” (85% confidence) based on its differential profile against the main cluster. (**c**) Similarly, two subpopulations of ductal cells were selected for comparative analysis. (**d**) The AI analysis reveals an unexpected functional state for the ductal subcluster (group1), predicting it as an “Activated state with ECM remodeling and antigen presentation” (85% confidence), suggesting a potential immune-related role.

This discovery of cells with intriguing dual functions, particularly combining tissue remodeling with immune activity, prompted us to delve deeper. However, the complexity of the solid tissue microenvironment made it difficult to determine whether these immune features were an intrinsic cellular property or a response to local cues. To isolate and characterize the intrinsic inflammatory state of such cells, free from confounding environmental factors, we first turned our attention to a dataset of peripheral blood mononuclear cells (PBMCs) (Figure 4).

#### 3.2.2. Assisted Discovery of Rare Activated NK Cell Subsets in PBMC Datasets

To investigate the unique functions of rare outlier cells within a known cell type, we identified and analyzed a small subset comprising just seven cells in the PBMC dataset. Using scSelector’s interactive lasso tool, we precisely delineated these seven cells, along with a larger cluster of the same cell type to serve as a control group. The system’s automated differential gene expression analysis revealed that these outlier cells had upregulated expression of *SLCO4A1*, *CTNNB1*, and *FPR2* [35] and downregulated expression of NPHS1 and LRRC8D. Functional enrichment analysis further demonstrated their enrichment in intracellular signaling cascades, vesicle-mediated transport, and cell activation pathways. Based on this molecular profile, the AI analysis defined their state as an “activated and stress-induced inflammatory state” with 90% confidence, characterized primarily by strong inflammatory signaling and enhanced vesicle-mediated transport activity.

However, while analyzing PBMCs provided valuable insights into the inherent inflammatory potential of these cells, their limitations quickly became apparent. As a circulating population, PBMCs cannot capture the complex, coordinated signaling and cell–matrix interactions that occur within a structured organ. To bridge this gap and explore how functionally diverse cells are orchestrated within a complex in vivo setting, we subsequently extended our research to a liver dataset. The liver, with its unique blend of metabolic and immune functions, provides the ideal platform to study this intricate cellular communication (see Supplementary Materials, Files S1 and S2).

#### 3.2.3. Assisted Discovery of Immune-Activated Cells and Metabolism-Related Cells in Liver Datasets

In the liver dataset, we first identified a group of outlier mature hepatocytes exhibiting prominent signaling characteristics. These cells showed high expression of multiple signaling-related genes, such as *SBK3*, *BRINP2*, *OPRL1*, and *GPR137B*, with functional enrichment highly concentrated in pathways related to intercellular signaling and ion channel activity. The AI analysis described their state as an “activated state with signaling capabilities” (85% confidence). These cells displayed features similar to neuroendocrine cells and may act as “information relay stations” within the liver microenvironment, coordinating the functional activities of various cell types.

However, an analysis of signal transduction alone is insufficient to explain the regulatory mechanisms of specific immune responses in the liver. Considering the liver’s role as a major immune organ, it is likely that specialized immune cell subsets are involved in its unique defense reactions. This prompted us to further analyze immune-related outlier cells within the liver dataset.

In another group of outlier cells, we identified significant immune features: high expression of immune-related genes, such as CD22 and DHPS [36], coupled with the downregulation of circulatory system-related genes, like F13A1, ANGPT1, and REN. Functional enrichment analysis confirmed a significant enrichment in immune pathways, including leukocyte differentiation. The AI model determined their state to be an “activated immune-related state with potential pro-inflammatory components, undergoing leukocyte differentiation” (85% confidence). This finding reveals a potential dynamic process where immune cells in the liver may shift from vascular-related functions to a state of immune activation, offering a new perspective on understanding liver-specific immune responses.

Although we had successfully resolved various functional states, some outlier cells still exhibited extremely complex or even seemingly contradictory molecular features that are difficult to explain using traditional functional frameworks. To test scSelector’s ability to address such challenging problems, we analyzed the most unique group of outlier cells in the liver dataset. This cell cluster showed an atypical expression pattern. On one hand, these cells displayed active lipid metabolism features (high expression of ACOT6 and ABCG1) [37]; on the other hand, their functional enrichment pointed significantly towards nervous system-related pathways, such as behavior, neurogenesis, and synaptic signaling. scSelector’s AI analysis system successfully summarized their state as a “metabolic stress response state regulated by neurogenesis” (85% confidence). This provided a biologically plausible explanation for the seemingly contradictory phenotype. These cells may be responding to specific microenvironmental stress by co-opting signaling pathways typically associated with the nervous system to coordinate metabolic reprogramming [38] (see Supplementary Materials, Files S1 and S2).

### 3.3. scSelector-Assisted Discovery of Cellular Subpopulation Physiological States and Biological Functions

The dynamic nature of intracellular gene expression results in cell type definitions that are not always rigidly defined, with transitional cell states (e.g., differentiation intermediates or activation phases) frequently observed. Additionally, clustering algorithms inherently exhibit limitations, as divergent clustering parameters often yield substantially different cluster quantities. Notably, cells of the same type may be partitioned into adjacent subclusters with close relationships, and functional annotation of these subclusters is important for elucidating the functional heterogeneity within specific cell populations.

#### 3.3.1. scSelector Reveals Functional Heterogeneity Within Pancreatic α-Cells

To validate scSelector’s capability in resolving functional heterogeneity within defined cell populations, we focused on α-cell clusters identified in a pancreatic single-cell dataset. Although traditionally annotated as a homogeneous population, clustering patterns revealed distinct subpopulations within α-cells. To investigate the functional significance of this observed heterogeneity, we employed scSelector’s interactive lasso tool to isolate a specific subcluster (termed Alpha-Sub1) for comparative analysis against the remaining α-cells.

Differentially expressed gene (DEG) analysis highlighted Alpha-Sub1’s unique molecular signature. Notably, genes such as *PLAC8* and the Notch ligand *JAG2* were significantly upregulated [39], while tumor suppressors, like *SMARCB1*, showed marked downregulation. Enrichment analysis confirmed this functional profile, with significant pathways including “External encapsulating structure,” “Cell adhesion,” “response to injury,” and “tissue morphogenesis,” indicating roles beyond classical endocrine functions. Leveraging scSelector’s integrated AI system, Alpha-Sub1 was classified with 90% confidence as an “activated, remodeling, and interaction-primed state.” Key features identified included ECM remodeling (supported by upregulation of FN1), signaling activation (implied by *JAG2*), and immune interaction potential (indicated by increased FCGR1A) [40]. These molecular signatures delineate an α-cell subpopulation potentially engaged in tissue repair and pathological processes, such as fibrosis.

To further explore this α-cell heterogeneity, we analyzed another distinct subcluster, Alpha-Sub2. Comparative analysis revealed a unique molecular profile, with DEG analysis showing significant downregulation of the Wnt pathway inhibitor *CXXC4* [41], alongside marked upregulation of *RANBP10* and *DGKH* [42], suggesting activation of pathways related to tissue repair and inflammatory responses. Functional enrichment analysis corroborated this, with top pathways including “cell adhesion,” “response to injury,” and “wound healing.”

**Figure 5:**
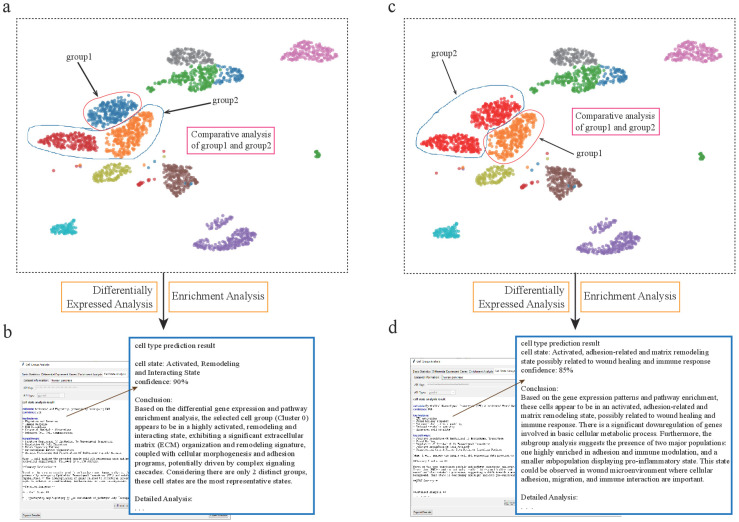
scSelector reveals functional heterogeneity within pancreatic alpha cell subpopulations. (**a**) UMAP visualization of the human pancreas dataset, highlighting heterogeneity within alpha cells, which form distinct subpopulations. The blue subcluster (group1) is selected for comparative analysis against the other alpha cells (group2). (**b**) The AI analysis output for the subcluster in (**a**) (corresponding to Alpha-Sub1 in the text), predicting its functional state as an “Activated, Remodeling and Interacting State” with 90% confidence. (**c**) In a second analysis, the orange/red alpha cell subcluster (labeled group1) is selected for comparison against the remaining alpha cells (group2). (**d**) The corresponding AI analysis for the subcluster in (**c**) (corresponding to Alpha-Sub2 in the text), predicting its state as an “Activated, adhesion-related and matrix remodeling state possibly related to wound healing and immune response” with 85% confidence.

scSelector’s AI system provided a detailed interpretation functional interpretation, defining Alpha-Sub2’s state as “an activated, adhesion- and matrix remodeling-associated state, potentially involved in wound healing and immune response” (85% confidence). The AI’s detailed interpretation highlighted its active matrix remodeling (e.g., elevated COL3A1, CHST11) and immune regulatory potential. Intriguingly, the AI noted the downregulation of basal metabolic genes (e.g., ATP5J2), suggesting a reallocation of cellular resources toward specialized repair functions. It also identified internal heterogeneity, with approximately 19% of the cells displaying more pro-inflammatory traits. These findings challenge the classical view of α-cells as solely glucagon-producing entities, demonstrating the existence of functionally heterogeneous α-cell subpopulations with the capacity for tissue repair and immunomodulation, which could be important during pancreatic injury or inflammation (Figure 5).

#### 3.3.2. scSelector Reveals a Neural Signaling-Specialized Mesenchymal Subpopulation in the Liver

To demonstrate the utility of scSelector across different tissues and cell types, we investigated the functional heterogeneity within the mesenchymal cell population of a liver dataset. Despite sharing a common “mesenchymal” annotation, UMAP visualization clearly segregated these cells into two distinct subpopulations. We utilized scSelector’s lasso tool to focus on one of these subpopulations (Mesenchymal-Sub1) and performed a comparative analysis against the other.

DEG analysis revealed the distinct molecular signature of Mesenchymal-Sub1. Key downregulated genes included *TPI1* and *CTNS* [43], whereas genes such as *HMSD* and C6orf132 were significantly upregulated. Notably, several genes associated with neural signaling, such as *KCNIP1* and GNB3, were markedly upregulated. Concurrently, the strong downregulation of *TPI1*, a key glycolytic enzyme, indicated significant metabolic reprogramming within this subpopulation.

Functional enrichment analysis strongly supported a specialized, non-classical function for Mesenchymal-Sub1. The most significantly enriched pathways were related to transcriptional regulation and neural processes, including “DNA-binding transcription factor activity,” “cell-cell signaling,” and “synaptic signaling.” Furthermore, pathways such as “neurotransmitter transport” and “monovalent inorganic cation transport” were also highly enriched. This enrichment pattern is highly atypical for hepatic mesenchymal cells, suggesting a unique functional specialization.

To obtain a detailed interpretation of this unusual expression profile, we leveraged the scSelector AI analysis system. Mesenchymal-Sub1 was defined as a “neural signaling and ion transport-specialized state with high transcriptional activity” (confidence: 85%). The AI analysis identified several key features defining this state. Its high transcriptional activity was indicated by enriched transcription-related pathways and a large number of DEGs, while its specialized neural signaling function was supported by the upregulation of neural-related genes and the enrichment of synaptic signaling pathways. Furthermore, the analysis pointed to significant ion transport activity, reflected in multiple enriched pathways, and a concurrent downregulation of basal metabolism, evidenced by the strong downregulation of *TPI1*. The AI analysis highlighted the rarity of such a prominent neural signaling signature within the context of hepatic mesenchymal cells. This finding is biologically significant, indicating the presence of a mesenchymal subpopulation in the liver with unexpected functional capabilities. This state may represent an aberrant differentiation, an adaptive response to specific stimuli (such as injury or inflammation), or a specialized cell type involved in processes like liver regeneration or intercellular communication within the hepatic microenvironment (see Supplementary Materials, Files S1 and S2).

### 3.4. scSelector-Assisted Discovery of Biological Functions in Cells Discarded During Preprocessing

Standard quality control (QC) procedures in single-cell RNA sequencing analysis often discard a fraction of cells presumed to be of low quality. However, the potential biological significance of these discarded populations is typically overlooked by conventional analysis workflows. For instance, when processing a human pancreas dataset, we observed that a large number of acinar cells were removed during the preprocessing step. We utilized scSelector to re-analyze this filtered acinar cell population to investigate whether it possessed specific biological functions.

DEG analysis revealed a unique molecular profile for this cell group. Genes such as *CSAD*, *HLA-DOA*, and *ITGB1* were significantly upregulated, while genes related to cell proliferation and specific signaling pathways, such as PRR23C, CEP55, and TGFBR2, were significantly downregulated. Consistent with these findings, functional enrichment analysis showed significant enrichment in pathways including “cell adhesion,” “DNA-binding transcription factor activity,” and “collagen-containing extracellular matrix.” Together, these results suggested that this cell population might be in a specialized functional state characterized by high adhesion, active transcriptional regulation, and matrix interaction capabilities.

To obtain a detailed interpretation functional assessment, we used the integrated AI analysis module of scSelector. Based on the aforementioned DEG and enrichment results, the model classified the state of these cells as “an activated state with strong cell adhesion, extracellular matrix remodeling, and immune interaction capabilities” with 90% confidence. Its reasoning was based on several key pieces of evidence: the cell adhesion and ECM remodeling capability supported by the upregulation of ITGB1 and related pathways; the potential immune interaction function suggested by the high expression of *HLA-DOA*; and a state of transcriptional activation confirmed by the upregulation of transcription factors such as PAX3 [44].

**Figure 6:**
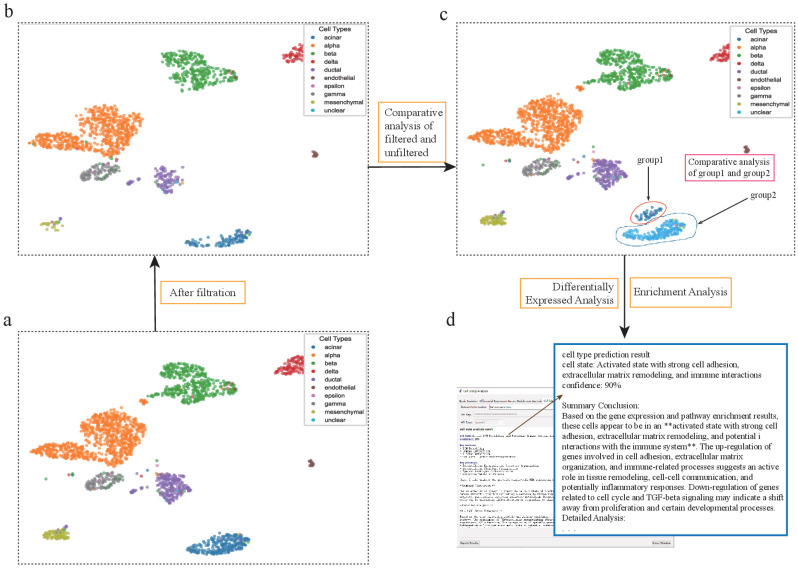
scSelector reveals biological functions in pancreatic acinar cells discarded by QC filtering. (**a**) UMAP visualization of the unfiltered human pancreas dataset prior to quality control (QC). (**b**) The same dataset after applying standard QC filtering, which resulted in the removal of a large population of acinar cells. (**c**) scSelector is used to perform a comparative analysis between the acinar cells that were discarded during QC (group1, circled) and those that were retained (group2). (**d**) The AI analysis output for the discarded cells (group1), predicting their state as an “Activated state with strong cell adhesion, extracellular matrix remodeling, and immune interactions” (90% confidence), suggesting they represent a biologically functional subpopulation rather than mere technical artifacts. (The symbol ‘**’ refers to Markdown formatting used for text emphasis.).

This analysis strongly suggests that a portion of the acinar cells filtered out by standard QC procedures may not be mere technical artifacts but rather a cell subpopulation with specific molecular characteristics and biological functions. This result highlights the value of re-evaluating discarded cell populations in single-cell analysis. By providing interactive selection and analysis functionalities, scSelector offers researchers an effective toolset to explore these often-ignored cell populations, facilitating a more detailed interpretation and understanding of cellular heterogeneity within a sample (Figure 6).

## 4. Discussion

### 4.1. Overcoming Core Limitations in Standard Workflows

Standard analysis workflows of scRNA-seq data face persistent challenges, particularly in balancing data quality filtering with the preservation of rare or unique cell states and in overcoming the limitations imposed by subjective clustering methods. This study introduces scSelector, an interactive toolkit designed to address these gaps by providing researchers with direct control over cell population selection and analysis, guided by their biological expertise.

A key challenge highlighted by Ilicic et al. (2016) [9] and Luecken & Theis (2019) [10] is the potential loss of biologically significant cells during stringent quality control. Traditional filtering often removes outliers without distinguishing technical artifacts from genuine, albeit rare or stressed, cellular states. scSelector directly tackles this by enabling users to visualize all cells, including potential outliers, and utilize its interactive lasso tool to select any population for downstream analysis, irrespective of QC flags or initial clustering results. Our results demonstrate the power of this approach. scSelector identified and characterized known rare cell types, like platelets in PBMCs, and extremely low-abundance endothelial cells in liver tissue, populations that could easily be overlooked or discarded by automated pipelines. This preserves valuable biological information and allows for the investigation of potentially important minority populations.

Furthermore, scRNA-seq analysis heavily relies on unsupervised clustering, a process fraught with subjectivity regarding parameter choices (e.g., cluster number), which significantly impacts downstream interpretation (Kiselev et al., 2019; Kharchenko et al., 2014) [11,12]. scSelector circumvents the rigidity of predefined clusters. By allowing users to select arbitrary groups of cells directly on the UMAP projection, it facilitates analysis tailored to specific biological hypotheses or visual patterns, rather than being constrained by algorithmic partitioning. This was important in dissecting functional heterogeneity within established cell types, such as the pancreatic α-cells. Standard clustering might group these cells together, masking subtle but important functional differences. scSelector enabled the isolation and comparison of specific α-cell subclusters, revealing distinct states associated with extracellular matrix remodeling, wound healing, and immune modulation—insights potentially missed by cluster-centric analysis. The ability to manually reposition cells or remove contaminating cells from a selection further enhances the precision of user-guided analysis.

### 4.2. A Researcher-Centric Paradigm with AI-Assisted Interpretation

The complexity of cellular relationships, especially in development or disease, often requires expert interpretation beyond algorithmic output (Tritschler et al., 2019; Saelens et al., 2019) [13,14]. scSelector is built around this principle, positioning the researcher as central to the analysis process. The tool provides the means (lasso selection, DEG analysis, enrichment) for investigators to explore populations based on visual cues or prior knowledge. The analyses of various outlier populations across datasets exemplify this, including identifying activated NK cells, secretory α-cells with ECM remodeling capacity, signaling hepatocytes, immune-activated liver endoderm cells, and metabolically stressed endothelial cells. These nuanced functional states were uncovered through targeted, user-driven investigation facilitated by scSelector.

To further aid interpretation, scSelector integrates large language models (LLMs) via API calls (DeepSeek, Gemini). The LLMs assist in predicting cell types and functional states based on the DEGs and enrichment results derived from user-selected populations. As shown across multiple examples, the LLM predictions, combined with confidence scores and reasoning, provide valuable hypotheses and summaries that researchers can critically evaluate against the molecular evidence and their own expertise. This synergistic approach combines the computational effectiveness of LLMs with the essential judgment of the biologist.

### 4.3. Contextual Positioning, Limitations, and Future Outlook

Compared to comprehensive pipelines, like Seurat and Scanpy, scSelector focuses specifically on enhancing the interactive exploration and refinement phase of analysis. While Seurat/Scanpy offers broader functionality, scSelector provides greater flexibility for defining and analyzing arbitrary cell groups independent of prior clustering. It complements annotation-focused tools (SingleR, CellAssign) and LLM-based methods (scGPT, scBERT) by providing an interactive interface to select the cells whose identity or function needs investigation.

Limitations of scSelector include potential scalability challenges for the interactive visualization component with extremely large datasets (millions of cells), though its reliance on Scanpy for backend processing is efficient. To quantitatively assess this efficiency, we have provided a detailed summary of the runtime and memory usage for the analyzed datasets in the Supplementary Materials (see the Supplementary Materials for details). The analysis is also dependent on the quality of the 2D embedding (UMAP), which can sometimes distort cellular relationships. Furthermore, while LLM integration is a useful feature, predictions are sensitive to the quality of input data and the inherent limitations of current AI models, necessitating careful validation by the researcher. The shift from clustering subjectivity to selection subjectivity is acknowledged, but we argue that researcher-guided selection based on visualization and biological context is a more informed approach. To validate the reliability of this approach, we assessed the tool’s robustness to user-to-user variability. Our results demonstrated that despite minor variations in the precise boundaries drawn by different users, the downstream LLM-based interpretation remained consistent across all replicates (see the Supplementary Materials for detailed results). This indicates that the core biological signal captured by scSelector is robust, and the integration of AI-assisted interpretation effectively buffers against minor manual selection jitters, ensuring reproducible biological conclusions.

Future developments could include integration with 3D visualization, support for trajectory inference analysis on selected populations, extensions for multi-modal data, and refined LLM prompting strategies.

In summary, scSelector provides a flexible and intuitive platform that enables researchers to move beyond the constraints of standard automated workflows. By integrating interactive cell selection with efficient backend analysis and AI-assisted interpretation, it facilitates the discovery of rare cell types, the characterization of outlier cell functions, and the dissection of functional heterogeneity within cell populations, ultimately enabling more detailed biological insights from single-cell data.

## 5. Conclusions

This study introduced scSelector (v1.0), an interactive toolkit designed to address limitations in current single-cell RNA sequencing data analysis workflows. By combining a user-friendly visualization interface with effective analytical functions and large language model integration, scSelector enables researchers to directly select and investigate cell populations of interest in real-time, guided by their expert knowledge. Key advantages include the ability to retain and analyze outlier cells often discarded by standard filtering, the flexibility to define cell groups independent of subjective clustering results, and the capacity to dissect functional heterogeneity within complex cell populations. Our results using diverse public datasets demonstrate scSelector’s effectiveness in identifying rare cell types (platelets, endothelial cells), characterizing distinct functional states (e.g., proliferative NK cells, activated secretory α-cells, signaling hepatocytes), and uncovering subtle subcluster differences within broader cell types (e.g., wound-healing-associated α-cells). We acknowledge, however, that the current version does not include built-in batch correction algorithms; therefore, when analyzing multi-batch or noisy clinical datasets, we recommend that users perform data integration using standard upstream pipelines prior to importing embeddings into scSelector to ensure robust interpretation. By placing researcher expertise at the center of the analysis process while leveraging computational tools and AI assistance, scSelector enhances the precision and flexibility of scRNA-seq analysis, offering a valuable approach for uncovering novel biological insights and exploring the complexity of cellular systems.

## Figures and Tables

**Table 1:** Packages used by scSelector.

Package	Version	Function
scanpy [2]	1.9.0	Toolkit for single-cell RNA-seq data analysis.
matplotlib [15]	3.4.0	Library for creating static, animated, and interactive plots.
numpy [16]	1.20.0	Fundamental package for numerical computing with Python.
gseapy [17]	0.14.0	Package for Gene Set Enrichment Analysis (GSEA).
pandas [18]	1.3.0	Library for data manipulation and analysis (DataFrames).
anndata	0.8.0	Annotated data structure for genomics data (esp. single-cell).
h5py	3.6.0	Pythonic interface to the HDF5 binary data format.
scikit-learn [19]	1.0.0	Comprehensive library for machine learning in Python.
statsmodels	0.13.0	Library for estimating and testing statistical models.
umap-learn [20]	0.5.0	Implementation of the UMAP dimensionality reduction algorithm.
celltypist [21]	1.1.0	Tool for automated cell type annotation or prediction.
requests	2.25.0	A simple and elegant library for sending HTTP requests in Python.

## Data Availability

The scSelector software developed in this study is open source and freely available on GitHub at https://github.com/miao1332211/scSelector (accessed on 11 December 2025). The public datasets analyzed during the current study are available in the Gene Expression Omnibus (GEO) repository. These include the human PBMC dataset (accession no. GSE132044) [24], the human pancreas dataset (accession no. GSE85241) [25], and the human liver dataset (accession no. GSE81252) [26]. The original contributions presented in the study are included in the article/Supplementary Materials, and detailed software metadata and technical specifications can be found in File S5. Further inquiries can be directed to the corresponding author.

## Supplementary Materials

The following supporting information can be downloaded at: https://www.mdpi.com/article/10.3390/genes17010002/s1. File S1: Differentially Expressed Genes (DEGs) and Enrichment Analysis; description of data: This file contains the detailed results of differentially expressed genes and functional enrichment analysis for each comparison group described in the Section 3; File S2: AI Analysis Results; description of data: This file contains the complete outputs and detailed reasoning from the large language model for the AI-assisted predictions for each analysis group described in the Section 3 and Table 1; File S3: Benchmarking of Differential Expression Methods and Computational Performance; description of data: This file contains the quantitative comparison results of the Mann–Whitney U test, Welch’s T-test, and DESeq2 regarding gene detection stability in rare populations, as well as the runtime and resource usage statistics for scSelector on the analyzed datasets; File S4: Robustness Analysis of User-Guided Selection and LLM Interpretation; description of data: This file contains the detailed results of the user-to-user variability experiment, including the selection metrics (cell counts and boundaries) from 10 independent users targeting the same cluster, along with the corresponding LLM prediction outputs for each replicate, demonstrating the consistency of biological interpretations despite minor variations in manual selection; File S5: Software Metadata and Technical Specifications for scSelector. Description of data: This file provides the project metadata for scSelector, including the project home page, supported operating systems (Windows, macOS, and Linux), and the programming language (Python). It also specifies requirements such as Anaconda/Miniconda for environment management, the necessity of DeepSeek or Gemini API keys for AI-powered functions, and the MIT License terms. There are no restrictions for use by non-academics.

## Abbreviations

The following abbreviations are used in this manuscript:

adj.*p*	Adjusted *p*-values
API	Application Programming Interface
BP	Biological Process
CC	Cellular Component
DEGs	Differentially Expressed Genes
GEO	Gene Expression Omnibus
GO	Gene Ontology
GSEA	Gene Set Enrichment Analysis
HVGs	Highly Variable Genes
KEGG	Kyoto Encyclopedia of Genes and Genomes
KNN	K-Nearest Neighbors
LLM	Large Language Model
log_2_FC	log_2_(Fold Change)
LSECs	Liver Sinusoidal Endothelial Cells
MF	Molecular Function
NK	Natural Killer
PBMC	Peripheral Blood Mononuclear Cell
PCA	Principal Component Analysis
QC	Quality Control
scRNA-seq	Single-cell RNA sequencing
UMAP	Uniform Manifold Approximation and Projection
