# Supplementary material for: scSelector: A Flexible Single-Cell Data Analysis Assistant for Biomedical Researchers
Source: Genes (Basel). 2025 Dec 19;17(1):2. doi: 10.3390/genes17010002 (PMC12841117; doi:10.3390/genes17010002)
Supplement: Supplementary file 1 [file genes-17-00002-s001.zip › Supplementary File S2.pdf]

Ai Analysis Results

3.1 scSelector-Assisted Annotation of User-specified Cell Clusters

3.1.1 Identification and Validation of the Platelet Cell Cluster

cell type prediction result

predicted cell type: Platelets confidence: 95%

Okay, I will analyze the provided single-cell RNA sequencing data to predict the most likely cell type for cluster 9.

Conclusion:

Based on the gene expression profile and pathway enrichment analysis, the most likely cell type for cluster 9 is **Platelets** with a confidence of **95%**.

Detailed Analysis:

1. **Up-regulated Gene Analysis:**
    - **Key Platelet Markers:** The most significantly up-regulated genes are strongly indicative of platelets. These include:
        - PF4 (Platelet Factor 4): A chemokine released from platelets during activation.
        - PPBP (Pro-Platelet Basic Protein, also known as CXCL7): Another chemokine released from activated platelets.
        - GP9 (Glycoprotein IX): A component of the GPIb-IX-V complex, a major platelet adhesion receptor.
        - CD9: A tetraspanin protein highly expressed on platelets, involved in platelet activation and aggregation.

    - TUBB1 (Tubulin Beta 1 Class VI): Found in megakaryocytes and platelets.
    - ITGA2B (Integrin Subunit Alpha 2b, also known as CD41): Forms a complex with ITGB3 (CD61) to form the GPIIb/IIIa receptor, a critical mediator of platelet aggregation.
    - GP9 (Glycoprotein IX) A member of the GPIb-IX-V complex.
    - SPARC (Secreted Protein Acidic And Rich In Cysteine) Associated with platelet aggregation.
  - **Other Supporting Genes:** Other upregulated genes like SDPR, GNG11, NRGN, CLU, CA2, F13A1, PTGS1, MPP1, TPM4, CLEC1B, ACTN1, CTSA, ILK, ESAM, and PARVB are also associated with platelet function, megakaryocytes, or hemostasis.
2. **Down-regulated Gene Analysis:** Since you haven't provided the list of significantly down-regulated genes, I cannot comment on their contribution to the cell type identification. Down-regulated genes can help exclude other potential cell types present in PBMCs.
3. **Pathway Enrichment Analysis:**
  - **Key Terms:** The top enriched pathways point towards platelet identity and function.
    - GOCC_CATALYTIC_COMPLEX: While broad, many components within this complex may be relevant to platelet signaling and metabolism. F13A1 for instance is involved in coagulation. GNG11 is a G-protein subunit.
    - GOCC_RIBOSOME, GOCC_RIBOSOMAL_SUBUNIT, and GOMF_STRUCTURAL_CONSTITUENT_OF_RIBOSOME: Platelets are known to contain ribosomes, although their role in protein synthesis within platelets is still under investigation. These pathways can be involved in the biogenesis of platelet proteins.
    - GOBP_INTRACELLULAR_TRANSPORT: Important for the trafficking of granules and other components within platelets.
    - GOCC_MITOCHONDRION: Platelets contain mitochondria, vital for energy production.
    - GOBP_ORGANONITROGEN_COMPOUND_BIOSYNTHETIC_PROC

ESS: This pathway could reflect the production of small molecules relevant to platelet activation and signaling.

- GOBP_CYTOPLASMIC_TRANSLATION: Reflects the platelet's capacity for protein synthesis, even though limited, and indicates that platelets are actively producing various components.

4. **Alternative Cell Type Considerations:**

   - **Megakaryocytes:** Megakaryocytes are platelet precursors. There could be some overlap in gene expression profiles, especially for genes involved in ribosome biogenesis and early platelet formation. However, the extremely high Log2FC values for definitive platelet markers such as PF4 and PPBP make this less likely.

5. **Dataset Background:**

   - **PBMCs:** Peripheral Blood Mononuclear Cells are a mixture of lymphocytes (T cells, B cells, NK cells), monocytes, and dendritic cells. The absence of strong markers for these other cell types, combined with the overwhelming presence of platelet markers, reinforces the conclusion that cluster 9 consists of platelets. Note that platelets are technically not *mononuclear*, but often co-purify during PBMC isolation.

3.1.2 Assist in Identifying the NK Cell Cluster in a Highly Proliferative State

cell type prediction result

predicted cell type: Activated cytotoxic lymphocytes (likely NK cells or CTLs) confidence: 90%

Okay, I will analyze the provided single-cell RNA sequencing data and predict the cell type of cluster 3.

Conclusion:

Based on the gene expression profile, pathway enrichment analysis, and the dataset background (human PBMC), the most likely cell type for cluster 3 is **activated cytotoxic**

lymphocytes, specifically likely a population of NK cells or cytotoxic T lymphocytes (CTLs). Confidence: 90%.

Detailed Analysis:

1. **Cluster Distribution and Differential Gene Expression:**
   - Cluster 3 consists of a small number of cells (7), suggesting it may be a rare cell type or a specific state within a more common cell type.
   - A large number of genes are differentially expressed (3707 upregulated, 2708 downregulated). This indicates a distinct transcriptional program compared to other cell clusters.
   - **Highly Upregulated Genes and Cell Type Association:**The most striking feature is the extremely high Log2FC values for genes like RRM2, KIAA0101, TYMS, TK1, MKI67, ZWINT, KIFC1, BIRC5, MYBL2, GINS2, PCNA, MCM3, MCM5, RRM1, HIST1H4C. These genes are strongly associated with cell cycle progression and proliferation. This suggests that the cells in cluster 3 are highly proliferative. Further supporting this is the upregulation of STMN1, which regulates microtubule dynamics during cell division.
   - **Cytotoxic Markers**: The presence of highly upregulated cytotoxic effector genes such as GZMA (Granzyme A), GZMH (Granzyme H), GZMK (Granzyme K), NKG7 and CST7 strongly indicates a cytotoxic lymphocyte population. Given the PBMC context, this points towards either Natural Killer (NK) cells or Cytotoxic T Lymphocytes (CTLs).
   - IFNG upregulation suggests these cells are activated and producing interferon-gamma, a key cytokine in immune responses and particularly important in NK and CTL function.
   - CXCR1 upregulation suggests that these cells can migrate to sites with high concentrations of IL-8 and other chemokines.
2. **Pathway Enrichment Analysis:**The top enriched pathways provide further insights:
   - GOBP_REGULATION_OF_IMMUNE_SYSTEM_PROCESS and GOBP_REGULATION_OF_IMMUNE_RESPONSE: Confirm the immune cell nature of these cells and their active role in modulating immune responses.

- GOBP_CELL_ACTIVATION, GOBP_LYMPHOCYTE_ACTIVATION and GOBP_REGULATION_OF_CELL_ACTIVATION: highlight these cells are in an activated state, which promotes cell signal transduction.
- GOBP_POSITIVE_REGULATION_OF_IMMUNE_SYSTEM_PROCESS: Reinforces the concept that these cells actively promote immune responses.
- GOBP_IMMUNE_RESPONSE_REGULATING_SIGNALING_PATHWAY: Indicates that these cells have the function of regulating the immune response through the cell surface receptor signaling pathway.
- GOBP_HEMOPOIESIS: It's quite common for immune cells to show enrichment in this pathway as they originate from the hematopoietic system. This pathway enrichment also is related with cell proliferation.
- GOBP_BIOLOGICAL_PROCESS_INVOLVED_IN_INTERSPECIES_INTERACTION_BETWEEN_ORGANISMS: The pathway is strongly associated with cell killing and cell activation process.
- GOBP_MONONUCLEAR_CELL_DIFFERENTIATION and GOBP_LEUKOCYTE_DIFFERENTIATION: Suggests that cells is experiencing differentiation process.

3. **PBMC Context:**Considering the dataset originates from PBMCs (Peripheral Blood Mononuclear Cells), the most likely candidates are NK cells and CTLs. Other possibilities would be activated B cells or T helper cells, but the cytotoxic gene signature is much stronger than what would be expected in those cell types.
4. **Distinguishing between NK cells and CTLs:** Differentiating between NK cells and CTLs solely based on this data is difficult without additional canonical markers. For example, absence of T cell markers like CD3D/E/G would favor NK cells, while presence of CD8A would support a CTL identity. The expression of both the Granzyme family and NKG7 suggests both cell types are activated. Some more specific markers would be needed for better resolution.
5. **Limitations:** Without information about the *downregulated* genes, I cannot give more support to the cell type conclusion.

3.1.3 Identification of Rare Endothelial Cell Clusters in Pancreatic Datasets

cell type prediction result

predicted cell type: Endothelial cells (likely microvascular endothelial cells) confidence: 95%

Okay, I've analyzed the provided single-cell RNA sequencing data. Here's my prediction and the reasoning behind it:

Conclusion:

Based on the gene expression profile and pathway enrichment, the most likely cell type for cluster 12 is **Endothelial cells (likely microvascular endothelial cells)** with a confidence of **95%**.

Detailed Analysis:

- **Up-regulated Genes and Cell Type Identification:**
    - The most significantly up-regulated genes (KDR, FLT1, PLVAP, SOX18, CALCRL, CD93, ESAM, EMCN, ELTD1, ERG, ACVRL1, PECAM1, CDH5, ROBO4, TEK, FLT4, ENG, PDGFB, etc.) are strong markers for endothelial cells. Many of these, such as *KDR* (VEGFR2), *FLT1* (VEGFR1), *PLVAP* (Plasmalemma Vesicle Associated Protein), *PECAM1* (CD31), *CDH5* (VE-cadherin), *ERG*, and *EMCN*, are well-established markers for endothelial cells across various tissues. *SOX18* is also known to play a key role in lymphatic endothelial cell development and angiogenesis. The co-expression of these genes at very high Log2FC values strongly points towards an endothelial cell identity. *ROBO4* is specifically expressed in endothelial cells and plays a role in angiogenesis. *FLT4* (VEGFR3) suggests possible lymphatic endothelial cell features.
    - The presence of *ACVRL1* (ALK1) and *ENG* (Endoglin) indicates the cluster might be involved in angiogenesis and vascular remodeling processes. *TEK* (also known as TIE2) is a receptor tyrosine kinase expressed predominantly by endothelial cells and hematopoietic stem cells. It plays a critical role in angiogenesis, vasculogenesis, and maintaining vascular integrity.

- **Pathway Enrichment Analysis:**
  - The pathway enrichment results provide further support for this identification. The top enriched GO terms are heavily related to endothelial cell functions:
    - **GOCC_ANCHORING_JUNCTION** and **GOCC_CELL_SUBSTRATE_JUNCTION:** These are critical for endothelial cell structure and interaction with the surrounding matrix. Endothelial cells form tight junctions that regulate permeability and maintain the integrity of the vasculature.
    - **GOBP_TUBE_DEVELOPMENT**, **GOBP_VASCULATURE_DEVELOPMENT**, **GOBP_TUBE_MORPHOGENESIS**, and **GOBP_BLOOD_VESSEL_MORPHOGENESIS:** These terms are directly related to the development and formation of blood vessels, a key function of endothelial cells.
    - **GOBP_ACTIN_FILAMENT_BASED_PROCESS**, **GOBP_CELL_MOTILITY**, **GOBP_LOCOMOTION**, **GOBP_AMEBOIDAL_TYPE_CELL_MIGRATION**: These terms are tightly related to the angiogenesis process, where endothelial cells need to proliferate and migrate to form new vessels.
    - **GOBP_REGULATION_OF_LOCOMOTION** and **GOBP_REGULATION_OF_INTRACELLULAR_SIGNAL_TRANSDUCTION**: Indicating the cells actively respond to signaling cues and remodel the cytoskeleton to allow cellular movement and adaptation to the environment.
    - **GOBP_CELL_CELL_JUNCTION**: Reinforces the cell type identification, as endothelial cells are the main component of blood vessels.
    - **GOBP_EPITHELIUM_DEVELOPMENT**, **GOBP_CELL_ADHESION**, **GOBP_REGULATION_OF_CELL_ADHESION** reinforces the adhesion function of endothelial cells in blood vessels.
- **Dataset Background Information (Human Pancreas):**
  - Considering the tissue source is the human pancreas, this cluster is likely composed of pancreatic endothelial cells. Pancreatic endothelial cells

play a crucial role in maintaining pancreatic function by providing blood supply and regulating interactions with other pancreatic cell types. It is likely to be microvascular endothelial cells due to the high levels of PLVAP expression.

- **Down-regulated Genes:** The provided results do not contain downregulated gene names, but it's important to know them to exclude cell types.
- **Alternative Cell Types and Reasons for Rejection:**
  - **Epithelial cells:** While some genes are shared with epithelial cells, the overwhelming enrichment of endothelial-specific markers makes this less likely.
  - **Fibroblasts:** Although fibroblasts are present in the pancreas, the endothelial marker expression is much stronger.
  - **Immune cells:** Given the gene markers and pathway enrichment results, the possibility of immune cell type is excluded.

3.1.4 Analysis and Prediction of Extremely Low-Abundance Endothelial Cell Clusters in Liver Datasets

cell type prediction result

predicted cell type: Liver Sinusoidal Endothelial Cells (LSECs) confidence: 95%

Okay, I will analyze the provided single-cell RNA sequencing data to predict the most likely cell type of cluster 9, considering gene expression, pathway enrichment, and dataset background information.

Conclusion:

Based on the highly upregulated marker genes (CLDN5, SLCO4A1, CDH5, PECAM1, etc.) and pathway enrichment analysis (Cell Adhesion, Plasma Membrane Region), the most likely cell type for cluster 9 is **Liver Endothelial Cells (LSECs)**. My confidence in this prediction is **95%**.

Detailed Analysis:

1. **Up-regulated Gene Analysis:**

- **CLDN5 (Claudin-5):** A well-established marker for endothelial cells, particularly in the liver. Its exceptionally high Log2FC (34.70) strongly suggests an endothelial origin.
- **SLCO4A1:** While not exclusively expressed in endothelial cells, it's known to be expressed in liver sinusoidal endothelial cells (LSECs) and plays a role in drug transport.
- **MMRN2 & MMRN1 (Multimerin 2 & 1):** Are secreted proteins associated with endothelial cells and involved in angiogenesis.
- **ABCG1:** ATP-binding cassette transporter G1, is also expressed in endothelial cells and involved in lipid transport.
- **IL33:** Interleukin 33, is expressed by endothelial cells and plays a role in immune modulation.
- **CALCRL:** Calcitonin Receptor-Like Receptor, expressed in endothelial cells and plays a role in vasodilation and angiogenesis.
- **BACE2:** Beta-Secretase 2, is also expressed in endothelial cells and involved in protein processing.
- **CDH5 (Cadherin-5/VE-Cadherin):** A crucial endothelial-specific adhesion molecule, essential for maintaining vascular integrity. The high Log2FC (34.42) reinforces the endothelial identity.
- **CD93:** An endothelial cell marker involved in phagocytosis and cell adhesion.
- **MGP:** Is also expressed in endothelial cells and involved in matrix remodeling.
- **PECAM1 (Platelet Endothelial Cell Adhesion Molecule-1/CD31):** Another key endothelial marker, involved in cell-cell adhesion and leukocyte transmigration. The very high Log2FC (34.36) further supports the endothelial identification.
- **ESAM:** Is also expressed in endothelial cells and involved in cell-cell adhesion.
- **ACKR1:** Is also expressed in endothelial cells and acts as a decoy receptor for several chemokines, modulating inflammatory responses.
- **VWF (von Willebrand Factor):** Synthesized by endothelial cells and

crucial for platelet adhesion and blood coagulation. A log2FC of 34.24 indicates a robust expression.

- **S1PR1 (Sphingosine-1-Phosphate Receptor 1):** Essential for endothelial cell survival and migration.
- **ERG:** Is a transcription factor that controls the expression of multiple endothelium-specific genes.
- **EGFL7:** Expressed by endothelial cells, is involved in angiogenesis and vascular development.
- **FLT1:** Is also expressed in endothelial cells and binds Vascular Endothelial Growth Factor (VEGF)

2. The collective expression of these highly upregulated genes provides strong evidence for the cells being endothelial in nature, and their specific combination points toward LSECs.
3. **Pathway Enrichment Analysis:**
    - **GOBP_CELL_ADHESION:** Highly significant, reflecting the adhesive properties of endothelial cells in forming the sinusoidal lining.
    - **GOCC_PLASMA_MEMBRANE_REGION:** Enrichment of plasma membrane related terms is expectable, as endothelial cells are located in vessel wall.
    - **GOBP_CELL_MOTILITY:** Endothelial cells needs to maintain cell motility during angiogenesis.
    - **GOBP_NEUROGENESIS:** LSECs can secrete neurotrophic factors that can promote neurogenesis.
    - **GOCC_COLLAGEN_CONTAINING_EXTRACELLULAR_MATRIX:** Liver sinusoidal endothelial cells (LSECs) in the liver play an important role in the synthesis and organization of ECM.
    - **GOBP_POSITIVE_REGULATION_OF_MULTICELLULAR_ORGANISMAL_PROCESS**: Related to cell development process.
    - **GOBP_SIGNALING_RECEPTOR_BINDING:** Endothelial cells has receptors that bind to signaling ligands.
    - **GOBP_REGULATION_OF_TRANSPORT:** LSECs involved in transporting small molecule.

- **GOBP_CELL_JUNCTION_ORGANIZATION:** Adherens junctions are very important to maintain cell cell interactions.
- **GOBP_POSITIVE_REGULATION_OF_DEVELOPMENTAL_PROCESS:** LSECs development
- **GOBP_RESPONSE_TO_WOUNDING**: The process of angiogenesis.
- **GOBP_ANATOMICAL_STRUCTURE_FORMATION_INVOLVED_IN_MORPHOGENESIS**: Cell and anatomical structure development
- **GOBP_CELL_CELL_SIGNALING:** LSECs can secret signals that regulate different cells.
- **GOBP_SECRETION:** LSECs can secret factors that promote different biological processes.
- **GOBP_TUBE_DEVELOPMENT**: Associated with blood vessel development.

4. These pathways consistently point toward the biological processes and structural features associated with endothelial cells, particularly those involved in vascular maintenance and regulation.
5. **Dataset Background Information (Human Liver):**
6. Considering the liver as the tissue source, the most relevant endothelial cell type would be liver sinusoidal endothelial cells (LSECs). LSECs are specialized endothelial cells that line the liver sinusoids, characterized by their unique fenestrated structure and roles in liver-specific functions like nutrient exchange, immune surveillance, and regulation of hepatic stellate cell activation.
7. **Alternative Cell Types and Reasons for Exclusion:**
    - **Other Endothelial Cell Types:** While other endothelial cell types exist in the liver vasculature (e.g., those lining larger blood vessels), the specific marker gene combination and high expression levels, especially CLDN5 and PECAM1, strongly favor LSECs.

3.2 scSelector-Assisted Discovery of Biological Functions in Outlier Cells

3.2.1 Assisted Discovery of Activated Cells with Extracellular Matrix Remodeling Function Under Mixed Conditions

part1

cell state analysis result

cell state: Activated Secretory State with ECM Remodeling Capacity confidence: 85%

key features: • ECM Remodeling • Cell Adhesion • Signaling and Secretion • Altered Metabolism

key pathways: • Extracellular encapsulating structure organization • Cell adhesion • Collagen containing extracellular matrix • Tube development • Cell-cell signaling • Cell Motility • Response to wounding

Okay, I will analyze the provided single-cell RNA sequencing data to infer the cell state, functional characteristics, and biological significance of the selected cell population (cluster 2). I will prioritize the analysis of gene expression and pathway enrichment results, and avoid making assumptions about the "cell type".

Summary Conclusion:

Based on the gene expression and pathway enrichment analysis, these cells (cluster 2) are most likely in a highly **activated and secretory state**, exhibiting strong extracellular matrix remodeling activity and cell adhesion capabilities, alongside potential involvement in tube/organ development. However, the downregulation of a few key metabolic genes needs to be considered. A high degree of ECM remodeling capability is observed.

Detailed Analysis:

1. **Cell State Inference:**

The cells of cluster 2 are characterized by a large number of upregulated genes, suggesting a highly **activated and dynamic state**. The enrichment of pathways related to "cell adhesion," "extracellular encapsulating structure," and "tube development" further supports this conclusion, indicating active cellular processes beyond a resting state. The presence of genes like *AURKB* and *NUSAP1*, which are related to cell cycle regulation, also suggests potential proliferative activity.

1. **Contribution of Up-regulated and Down-regulated Genes:**
    - **Up-regulated Genes:** The significantly upregulated genes point to the following features:
    - **Extracellular Matrix (ECM) Remodeling:** Many highly upregulated genes (e.g., *MMP10*, *COL6A6*, *COL4A2*, *ADAMTS* family members, *LOXL2*, *FN1*) are involved in ECM organization, degradation, and remodeling. This highlights a dynamic interaction between the cell and its surrounding environment. This ability to heavily remodel the extracellular matrix is likely a dominant feature of this cell state.
    - **Cell Adhesion and Interaction:** Increased expression of genes like *ITGA4*, *ITGAV*, *ROBO2*, *NCAM1*, and other cell adhesion molecules indicates a strong capacity for cell-cell and cell-matrix interactions. The cells are actively interacting with their environment and other cells.
    - **Signaling and Secretion:** Upregulation of signaling molecules (e.g., *WNT2*, *SHH*, *TGFB3*) indicates active intercellular communication, influencing developmental processes. Several upregulated genes are also involved in protein secretion or extracellular vesicle formation.
    - **Ion Channel Activity:** Upregulation of *CNGA4* and *KCNC3* hints towards modified ion channel function of these cells.
    - **Ribosomal proteins:** The upregulation of RPL9 and EIF family members suggests active protein translation
    - **Down-regulated Genes:** The downregulation of a small number of genes, such as *BHMT* (involved in methionine metabolism) and *COX7A2* (involved in mitochondrial respiration), suggests a possible shift in metabolic priorities or a general decrease in energy production by mitochondria, although the dramatic difference in up vs downregulation should be noted. This would be an interesting area of further exploration.
2. **Main Functional Characteristics:**

Based on the analysis, the main functional characteristics of these cells are:

* **ECM Remodeling and Organization:** Predominant activity involved in modifying the extracellular matrix.

* **Cell Adhesion and Motility:** Enhanced capacity for cell-cell and cell-matrix interactions, potentially related to migration or tissue organization.

* **Signaling and Communication:** Active intercellular communication via secreted factors.

* **Potential Proliferation:** Some indications of proliferative activity, but needs further validation.

* **Altered Metabolism:** Possible shifts in energy metabolism.

1. **Key Molecular Markers and Pathways:**

    - **Key Up-regulated Markers:** *GEMIN5, ARHGAP20, CNGA4, LOC728175, CDCA2, STX1B, TAS2R3, LINC00330, MAP1B, LOC400655, MMP10, COL6A6, COL4A2, ADAMTS family members, LOXL2, FN1*
    - **Key Down-regulated Markers:** *CTSL1P2, BHMT, COX7A2*
    - **Key Pathways:** "External Encapsulating Structure Organization", "Cell Adhesion", "Collagen Containing Extracellular Matrix", "Tube Development", "Animal Organ Morphogenesis", "Cell-Cell Adhesion", "Cell Motility"

2. **Significance of Cell State in Biological/Disease Context:**

The observed cell state, with its focus on ECM remodeling, cell adhesion, and signaling, could be significant in several biological and disease contexts:

* **Developmental Processes:** These activities are crucial for tissue formation, organogenesis, and cell migration during development.

* **Tissue Repair and Regeneration:** ECM remodeling and cell migration are key features of wound healing and tissue repair. The cells may be involved in orchestrating these processes.

* **Fibrosis:** Aberrant ECM remodeling and cell adhesion can lead to fibrotic conditions in various organs. These cells may be contributing to or actively involved in the fibrotic process.

* **Cancer:** ECM remodeling is a crucial step in cancer progression, allowing tumor cells to invade surrounding tissues and metastasize. The observed cell state might be related to tumor microenvironment remodeling.

* **Inflammation:** This activation state might be induced by inflammation, and these

cells may contribute to inflammatory responses through the secretion of signaling molecules.

part2

cell state analysis result

cell state: Activated state with ECM remodeling and antigen presentation confidence: 85%

key features: • ECM remodeling • Antigen presentation • Altered metabolic activity • Skeletal system involvement

key pathways: • GOCC_EXTERNAL_ENCAPSULATING_STRUCTURE • GOCC_COLLAGEN_CONTAINING_EXTRACELLULAR_MATRIX • GOMF_EXTRACELLULAR_MATRIX_STRUCTURAL_CONSTITUENT • GOBP_PEPTIDE_ANTIGEN_ASSEMBLY_WITH_MHC_CLASS_II_PROTEIN_COMPLEX

Okay, I will analyze the provided single-cell RNA sequencing data to determine the state and functional characteristics of the selected cell cluster. I will focus on interpreting the gene expression patterns and pathway enrichments without pre-defining a specific cell type.

Summary Conclusion:

Based on the gene expression profile and pathway enrichment analysis, these cells appear to be in an **activated state associated with extracellular matrix remodeling and antigen presentation**, possibly indicative of an immune-related function within the pancreas. There is also evidence of altered metabolic activity and skeletal system involvement.

Detailed Analysis:

1. Cell State:

The cells are most likely in an activated state rather than resting or quiescent. This activation is characterized by distinct molecular mechanisms, which I will delineate in the following sections. The enrichment in skeletal system development is intriguing and could indicate these cells are involved in tissue repair or remodeling process in pancreas microenvironment.

2. Contribution of Up-regulated and Down-regulated Genes:

- **Up-regulated Genes:** The most highly up-regulated genes paint a picture of cells deeply involved in immune interaction and extracellular matrix dynamics.
    - OPRL1, GPR137B, and GPR65 are G-protein coupled receptors (GPCRs). Upregulation of these markers indicates altered signal transduction and cellular responses to external stimuli.
    - ITGB2 (Integrin Subunit Beta 2): This gene is a critical component of leukocyte adhesion molecules and plays a crucial role in cell-cell and cell-matrix interactions, particularly in immune cell trafficking and activation. High expression suggests increased interaction with other cells, including immune cells.
    - HLA-DQA1 (Major Histocompatibility Complex, Class II, DQA1): This gene encodes a part of the MHC class II complex, which is crucial for antigen presentation to T helper cells. Its upregulation strongly suggests an antigen-presenting role for these cells.
    - CD84: This is a member of the SLAM family of immune receptors. Upregulation is suggestive of immune cell interactions.
    - Several matrix metalloproteinases (MMP14) and collagens (COL8A1)are significantly upregulated, indicative of the ECM remodeling
- **Down-regulated Genes:** The down-regulated genes point to a reduced proliferative capacity.
    - CCND1 and CCND2 (Cyclin D1 and Cyclin D2): These genes are key regulators of the cell cycle. Their down-regulation suggests that these cells are not actively proliferating.
    - Downregulation of NRAS indicate a reduced cell proliferation.

3. Main Functional Characteristics:

- **Extracellular Matrix Remodeling:** The strong enrichment of pathways related to the extracellular matrix (ECM), like "GOCC_EXTERNAL_ENCAPSULATING_STRUCTURE" and "GOCC_COLLAGEN_CONTAINING_EXTRACELLULAR_MATRIX," indicates an active role in remodeling the surrounding tissue. This could be due to inflammation, tissue repair, or even an invasive process.
- **Antigen Presentation:** The enrichment of "GOBP_PEPTIDE_ANTIGEN_ASSEMBLY_WITH_MHC_CLASS_II_PROTEIN_COMPLE

X" and the upregulation of HLA-DQA1 point to a role in presenting antigens, which is a key function of antigen-presenting cells (APCs) to activate T cells.

- **Altered Metabolic Activity:** Genes involved in organic acid and small molecule metabolic processes show significant expression changes, suggesting metabolic reprogramming. This could be related to the activation state and the energy demands of the cells.
- **Skeletal System Involvement:** Although seemingly out of context, the enrichment of "GOBP_SKELETAL_SYSTEM_DEVELOPMENT" could indicate involvement in tissue repair processes, as these developmental pathways are often reactivated in adult tissues during injury and regeneration.

4. Key Molecular Markers and Pathways:

- **Molecular Markers:**
  - *ECM Remodeling:* MMPs (e.g., MMP14, MMP19, MMP3, MMP28) and collagens (e.g., COL8A1, COL12A1, COL16A1)
  - *Immune Cell Activation/Interaction:* ITGB2, CD84, HLA-DQA1, SIGLEC5
  - *GPCR Signaling:* OPRL1, GPR137B, GPR65
- **Pathways:**
  - GOCC_EXTERNAL_ENCAPSULATING_STRUCTURE
  - GOCC_COLLAGEN_CONTAINING_EXTRACELLULAR_MATRIX
  - GOMF_EXTRACELLULAR_MATRIX_STRUCTURAL_CONSTITUENT
  - GOBP_PEPTIDE_ANTIGEN_ASSEMBLY_WITH_MHC_CLASS_II_PROTEIN_COMPLEX
  - GOBP_SKELETAL_SYSTEM_DEVELOPMENT

5. Biological or Disease Context:

In the context of the human pancreas, these cells could be:

- **Involved in local inflammatory responses:** The ECM remodeling and immune activation signatures are consistent with inflammation. This could be related to pancreatitis, diabetes (insulitis), or other inflammatory conditions affecting the pancreas.
- **Participating in tissue repair after injury:** The ECM remodeling and skeletal system development pathways could be activated during tissue repair processes

in the pancreas.

- **Interacting with the tumor microenvironment:** If this data is from a pancreatic tumor, these cells could be playing a role in the tumor microenvironment, either promoting or inhibiting tumor growth. The ECM remodeling could facilitate tumor invasion, while the antigen presentation could stimulate an anti-tumor immune response.
- **Contributing to the pathogenesis of autoimmune diseases:** The antigen-presenting function could be related to the development or progression of autoimmune diseases affecting the pancreas.

In summary, this cell population seems to be involved in active tissue remodeling, probably participating in ECM dynamics and inflammation. The exact role depends on the specific disease context within the pancreas, which is not specified here.

3.2.2 Assisted Discovery of Rare Activated NK Cell Subsets in PBMC Datasets

cell state analysis result

cell state: Activated and Stressed Inflammatory State confidence: 90%

key features: • High inflammatory response • Increased vesicle-mediated transport • Metabolic reprogramming • Regulation of T cell differentiation

key pathways: • Intracellular Signaling Cascade • Vesicle-mediated Transport • Response to Oxygen-containing Compound • Cell Activation • Immune System Process Regulation

Okay, I've reviewed the provided single-cell RNA sequencing data analysis results for this cell population derived from human PBMCs. Here's a detailed analysis of their state, function, and potential biological significance, followed by a JSON summary:

Conclusion:

These cells from Group 1 (7 cells within Cluster 3) exhibit a highly **activated and stressed state**, characterized by a strong inflammatory response signature, metabolic reprogramming towards increased nucleotide metabolism, and significant alterations in vesicle-mediated transport processes. The cell state reflects an urgent need for rapid response and immune regulation. The low number of cells makes subgroup analysis unreliable, so more data is necessary to draw any conclusions.

Detailed Analysis:

1. **Cell State (Activated & Stressed):**
    - The overwhelming number of up-regulated genes (4960) compared to down-regulated genes (633) indicates a significant shift in the cell's transcriptional program, pointing towards activation rather than inhibition or quiescence.
    - Enrichment in pathways like "GOBP_INTRACELLULAR_SIGNALING_CASSETTE," "GOBP_RESPONSE_TO_OXYGEN_CONTAINING_COMPOUND", "GOBP_CELL_ACTIVATION", "GOBP_REGULATION_OF_IMMUNE_SYSTEM_PROCESS", and "GOBP_INFLAMMATORY_RESPONSE" provides strong evidence for an active response to stimuli.
    - Downregulation of NPHS1 points to a state of cellular stress/dysfunction, as this gene is essential for maintaining glomerular filtration barrier integrity.
2. **Contribution of Up-regulated and Down-regulated Genes:**
    - **Up-regulated genes:** The highly up-regulated genes highlight the core functions activated in these cells:
        - SLCO4A1: Solute carrier organic anion transporter family member 4A1 expression suggests a role in the transport of small molecules, potentially related to nutrient uptake or removal of waste products during activation.
        - CTNNB1 (Beta-catenin): Upregulation of CTNNB1 indicating activation of the Wnt signaling pathway, this often occurs in developmental process, cell proliferation and differentiation.
        - ADAMTS4: A protease, might indicate extracellular matrix remodeling or shedding of surface proteins related to cell-cell interaction or signaling.
        - FPR2: A receptor involved in inflammatory responses, indicates a critical role in responding to inflammatory signals.
        - WNT1: Upregulation of WNT1 activates Wnt signaling, promoting cell proliferation and differentiation.

- **Down-regulated genes:**
    - ZNF621 and RP11-517P14.2: The function of these genes are relatively unknown.
    - LRRC8D: Involved in cell volume regulation and amino acid transport, suggesting a potential change in cell metabolism.
    - NPHS1: Downregulation may relate to cell stress.
    - PTGDS: Downregulation indicating a role in cell stress.

3. **Main Functional Characteristics:**
    - **Inflammatory Response:** A dominant theme. "GOBP_INFLAMMATORY_RESPONSE" is highly enriched, along with regulation of cell activation, and immune system processes.
    - **Vesicle-mediated Transport:** Significant enrichment of "GOBP_VESICLE_MEDIATED_TRANSPORT" and "GOBP_ENDOCYTOSIS" points to increased exocytosis/endocytosis, potentially to secrete cytokines, chemokines, or other signaling molecules, or to internalize receptors.
    - **Metabolic Reprogramming:** "GOBP_RESPONSE_TO_OXYGEN_CONTAINING_COMPOUND" suggests changes in cellular metabolism, possibly to meet the increased energy demands of activation.
    - **Regulation of T Cell differentiation:** GOBP_REGULATION_OF_CD4_POSITIVE_ALPHA_BETA_T_CELL_DIFFERENTIATION and GOBP_REGULATION_OF_ALPHA_BETA_T_CELL_DIFFERENTIATION is highly enriched.
4. **Key Molecular Markers and Pathways:**
    - **Molecular Markers:**
        - Up-regulated: SLCO4A1, CTNNB1, ADAMTS4, FPR2, WNT1.
        - Down-regulated: NPHS1
    - **Pathways:**
        - Intracellular Signaling Cascade

        - Vesicle-mediated Transport
        - Response to Oxygen-containing Compound
        - Cell Activation
        - Immune System Process Regulation
        - Leukocyte differentiation
5. **Significance in Biological/Disease Context:**
    - The state described is a hallmark of immune responses. These cells may be actively participating in an immune response to infection, inflammation, or even autoimmunity. The high level of activation and stress suggests a recent or ongoing encounter with a stimulus.
    - In a disease context, these cells could be contributing to pathology, such as in chronic inflammatory diseases, autoimmune disorders, or during the cytokine storm associated with severe infections.
6. **Subgroup Analysis:**
    - Given that there are only 7 cells from Cluster 3, and the comparison group is 145 cells from the same cluster, there is currently not sufficient evidence to meaningfully analyze subgroups *within* the current selected cell population.

3.2.3 Assisted Discovery of Immune-Activated Cells and Metabolism related cells in Liver Datasets

part1

cell state analysis result

cell state: Activated and Signaling-Competent confidence: 85%

key features: • Enhanced cell-cell communication • Neuroendocrine-like signaling • Regulation of membrane potential • Decreased basic metabolism

key pathways: • GOBP_CELL_CELL_SIGNALING • GOCC_MONOATOMIC_ION_CHANNEL_COMPLEX •

GOMF_GATED_CHANNEL_ACTIVITY • GOBP_SYNAPTIC_SIGNALING • GOBP_REGULATION_OF_MEMBRANE_POTENTIAL

Okay, I will analyze the provided single-cell RNA sequencing data to infer the state and function of the selected cell population, focusing on the implications of differential gene expression and pathway enrichment.

Summary Conclusion:

Based on the gene expression profile and enriched pathways, the selected cell population appears to be in a highly **activated and signaling-competent state**, characterized by enhanced cell-cell communication, ion channel activity, and synaptic signaling-related processes, and exhibiting some features of neuroendocrine function. The high confidence stems from the consistency of the enriched pathways and the function of differentially expressed genes. However, it is also characterized by decreased expression of some basic metabolism related genes, suggesting a shift away from basal metabolic activities.

Detailed Analysis:

1. **Cell State:**
    - **Activated:** The strong enrichment of pathways like "GOBP_CELL_CELL_SIGNALING" and "GOBP_SYNAPTIC_SIGNALING" indicates an active communication state, where cells are actively sending and receiving signals. The upregulation of *NTF4* (Neurotrophin-4) further supports this.
    - **Signaling-Competent:** The enrichment of pathways related to ion channel activity ("GOCC_MONOATOMIC_ION_CHANNEL_COMPLEX", "GOMF_GATED_CHANNEL_ACTIVITY") suggests that the cells are highly responsive to external stimuli and capable of generating rapid electrical signals.
2. **Contributions of Up-regulated and Down-regulated Genes:**
    - **Up-regulated Genes:**
        - The high Log2FC values for genes like *SBK3*, *BRINP2*, and *CYP2A13* suggest a significant shift in the cell's molecular machinery. *BRINP2* and *NALCN* are associated with neuronal function, which hint at a neuroendocrine-like capacity.
        - *LCN2* upregulation suggests potential inflammatory signaling.

        - Upregulation of genes involved in neurotransmitter receptors such as *CHRNA3* and *NOS1* indicates a role in signal transduction.
        - *ACLY* upregulation can be related to increased lipid biosynthesis and energy regulation.
    - **Down-regulated Genes:**
        - The strong downregulation of *PHKG1* indicates a decrease in glycogenolysis, implying a shift away from glucose metabolism.
        - Downregulation of *CFTR* suggests alteration in ion transport processes.

3. **Main Functional Characteristics:**
    - **Enhanced Cell-Cell Communication:** Supported by the enrichment of "GOBP_CELL_CELL_SIGNALING" and upregulation of signaling molecules and receptors.
    - **Neuroendocrine-like Signaling:** Enrichment in "GOBP_SYNAPTIC_SIGNALING", coupled with the upregulation of neuronal markers, suggest that these cells might be involved in neuroendocrine-like signaling even if they are not classic neuronal cells.
    - **Regulation of Membrane Potential and Ion Transport:** The enrichment of pathways related to ion channel activity, coupled with the differential expression of specific ion channel genes like *NALCN* and the downregulation of *CFTR*.
    - **Decreased Basic Metabolism:** Downregulation of *PHKG1*, *PGM5* suggest a lower rate of glycogenolysis, shifting away from the usual metabolism.

4. **Key Molecular Markers and Pathways:**
    - **Key Up-regulated Markers:** *SBK3, BRINP2, CYP2A13, NALCN, CHRNA3, NOS1, LCN2*
    - **Key Down-regulated Markers:** *PHKG1, CFTR*
    - **Key Pathways:** GOBP_CELL_CELL_SIGNALING, GOCC_MONOATOMIC_ION_CHANNEL_COMPLEX, GOMF_GATED_CHANNEL_ACTIVITY, GOBP_SYNAPTIC_SIGNALING,

GOBP_REGULATION_OF_MEMBRANE_POTENTIAL.

5. **Significance of the Cell State:**
6. The described cell state may represent a specialized or activated state within a tissue, potentially related to:
   - **Response to Stimuli:** The heightened signaling capacity may be a response to external cues (e.g., inflammation, stress).
   - **Tissue Repair or Remodeling:** Activated cells may play a role in tissue repair or remodeling processes by coordinating with neighboring cells and modulating the cellular microenvironment.
   - **Disease Pathogenesis:** In the context of disease, this state may contribute to the underlying mechanisms, potentially exacerbating inflammation or driving cellular dysfunction.

part2

cell state analysis result

cell state: Activated immune-related state with a potential pro-inflammatory component, undergoing leukocyte differentiation confidence: 85%

key features: • Leukocyte differentiation • Cytoskeletal motor activity • Altered cell adhesion • DNA binding transcription factor activity

key pathways: • GOBP_LEUKOCYTE_DIFFERENTIATION • GOBP_CIRCULATORY_SYSTEM_PROCESS • GOBP_CELL_ADHESION

Okay, I will analyze the provided single-cell RNA sequencing data to infer the biological state and functional characteristics of the selected cells (cluster 3). I will focus on interpreting the gene expression patterns and pathway enrichments without assuming the specific cell type.

Summary Conclusion:

Based on the provided data, the selected cells (cluster 3) appear to be in an **activated immune-related state, likely involved in leukocyte differentiation and potentially displaying a pro-inflammatory profile**. Key features include upregulated expression of immune signaling molecules, cytoskeletal motor activity and downregulation of genes

involved in circulatory system process. There's evidence of altered cell adhesion and DNA binding transcription factor activity. The confidence level for this conclusion is 85%.

Detailed Analysis:

1. Cell State Inference:

The enrichment analysis strongly suggests that these cells are actively involved in **leukocyte differentiation (GOBP_LEUKOCYTE_DIFFERENTIATION)**. The significant number of pathways related to developmental processes (e.g., animal organ morphogenesis, cell morphogenesis, cell projection morphogenesis) suggests these cells might be undergoing substantial changes in structure or shape, potentially related to their functional activation and differentiation. Also, there is high score for cytoskeletal motor activity.

The downregulation of GOBP_CIRCULATORY_SYSTEM_PROCESS suggests that these cells may be detaching or altering its interaction with the circulatory system, which is consistent with cell differentiation.

2. Contributions of Up-regulated Genes:

- **Immune Signaling and Activation:** Upregulation of CD22 (B-cell receptor), SH2D6 (SH2 domain containing 6), LILRB3 (Leukocyte Immunoglobulin Like Receptor B3), and several interferon-induced genes (e.g., IFNA1, IFNA5) strongly indicates an activated immune response. The high Log2FC of CD22 suggests a B-cell related function. MYD88 upregulation points to TLR signaling pathway activation. Upregulation of ZBTB7B (also known as THPOK), which is a transcription factor implicated in T cell development. It's involved in the differentiation and maintenance of CD4+ T cells.
- **Cellular Processes and Metabolism:** Upregulation of genes like MIOX (myo-inositol oxygenase), TRAP1 (TNF receptor-associated protein 1) which points to altered metabolic state and potentially increased stress response.
- **Cytoskeleton and Cell Motility:** The high number of genes involved in cytoskeletal motor activity could indicate change in cell motility, such as migration of differentiated cells.

3. Contributions of Down-regulated Genes:

- **Vascular/Circulatory System Processes:** The prominent downregulation of F13A1 (coagulation factor XIII A chain), ANGPT1 (angiopoietin 1) and REN (renin) strongly implies that these cells are disengaging from circulatory system function or losing features associated with vascular homeostasis.

Downregulation of these genes suggests the cells are changing their interaction with the local vascular environment.

- **Transcription factors and regulators:** The down-regulation of genes like ELF1 and NR4A2 suggests an shift in the transcriptional regulation program within these cells. NR4A2 is an immediate early gene. It encodes a member of the steroid/thyroid hormone receptor superfamily. As an inducible transcription factor, this protein plays a role in signal transduction and transcriptional regulation.

4. Main Functional Characteristics:

- **Immune Response and Inflammation:** Activation of immune signaling pathways (TLR, interferon) and leukocyte differentiation are prominent.
- **Altered Cell Adhesion:** Changes in cell adhesion are indicated by both up- and down-regulated genes related to cell-cell and cell-matrix interactions. This suggests the cells are undergoing changes to its local environment.
- **Metabolic Reprogramming:** Upregulation of genes related to metabolism (MIOX) and stress response (TRAP1) suggests that cells undergo metabolic switch.

5. Key Molecular Markers and Pathways:

- **Key Up-regulated Markers:**
  - CD22: B-cell receptor, suggests a B-cell related function.
  - DHPS: Involved in folate biosynthesis, also related to immune response.
  - CLCNKA: Chloride channel, potentially involved in cellular homeostasis during differentiation.
- **Key Down-regulated Markers:**
  - F13A1: Coagulation factor XIII, indicates reduced involvement in coagulation processes.
  - ANGPT1: Angiopoietin-1, suggesting reduced interaction with vascular cells.
  - REN: Renin, further supports disengagement from circulatory system function.
- **Key Pathways:**

- GOBP_LEUKOCYTE_DIFFERENTIATION: Strong evidence of active leukocyte differentiation.
- GOBP_CIRCULATORY_SYSTEM_PROCESS: Downregulation indicates cells are disengaging from circulatory system function.
- GOBP_CELL_ADHESION: Altered cell adhesion suggests cells undergo changes to its local environment.

6. Biological Significance:

The state of these cells could be relevant in several biological or disease contexts:

- **Inflammation:** An activated immune-related state could be indicative of a response to tissue damage or infection in the liver.
- **Disease Progression:** This state may also reflect pathological processes such as liver fibrosis or tumor microenvironment modulation, where immune cells and cytokines play a pivotal role.
- **Normal Immune Surveillance:** These cells could be involved in normal immune surveillance functions within the liver tissue.

part3

cell state analysis result

cell state: Neurogenesis modulated metabolic stress response state confidence: 85%

key features: • Enhanced lipid metabolism • Active modulation of neurogenesis and synaptic signaling • Suppressed cell-cell adhesion • Modulation of neuronal differentiation

key pathways: • GOBP_BEHAVIOR • GOBP_NEUROGENESIS • GOBP_SYNAPTIC_SIGNALING

Okay, I will analyze the provided single-cell RNA sequencing data for cluster 9 cells, focusing on their state and function, based on the differential gene expression and pathway enrichment results. I will avoid making assumptions about cell type identity and concentrate on the observed molecular features.

Summary Conclusion:

Cluster 9 cells appear to be in a highly dynamic and potentially stressed state characterized by enhanced metabolic activity (specifically lipid metabolism), active neurogenesis and synaptic signaling modulation, and suppression of cell-cell adhesion and certain neuronal development-related processes. This suggests a context where these cells are actively adapting or responding to internal or external stimuli, possibly related to neuronal remodeling or an attempt to maintain neuronal function under adverse conditions, such as inflammation or hypoxia. The down-regulation of genes involved in cell-cell adhesion, like some cadherins and integrins, implies a potential change in cell interaction within the human liver environment.

JSON Output:

Detailed Analysis:

1. Cell State Inference:

The enriched pathways and differentially expressed genes point toward a combination of a metabolic stress response intertwined with neurogenesis, and modulated synaptic signaling:

- **Dynamic State:** The strong up-regulation of genes involved in behavior, coupled with neurogenesis and synapse function, indicates a very active and potentially stressed neuronal state. The concurrent modulation of multiple key cellular processes suggests an active response to a changing environment.
- **Stress:** The specific up-regulation of certain genes in response to neurogenesis and synaptic signaling pathways indicates potential cellular stress, such as limited resources, hypoxia, or inflammation.

2. Contributions of Up-Regulated and Down-Regulated Genes:

- **Up-Regulated Genes:**
    - ACOT6 (Acyl-CoA Thioesterase 6): Extremely high Log2FC indicates a significant increase in fatty acid metabolism. ACOT6 hydrolyzes acyl-CoA, regulating fatty acid levels. This could be linked to energy production under stress or altered lipid metabolism.
    - ABCG1 (ATP Binding Cassette Subfamily G Member 1): Involved in cholesterol and lipid transport. Up-regulation can contribute to lipid homeostasis.
    - GKN2 (Gastrokine 2): Although its exact function isn't fully known, it's often found in association with stress response in epithelia.

- AURKA: Involved in the cell cycle and mitosis, which may indicate the cell is undergoing neurogenesis and cell differentiation.
- CRTC2: Involved in regulating metabolic genes, which is related to lipid metabolism.

- The upregulation of these genes indicates that the cell is in a state of enhanced metabolic activity, actively modulating synaptic signaling, and perhaps undergoing changes in structural protein composition.
- **Down-Regulated Genes:**
    - CHKB (Choline Kinase Beta): Involved in phospholipid biosynthesis. Down-regulation may affect membrane structure and signaling pathways.
    - FZD4 (Frizzled-4): Receptor in the Wnt signaling pathway, crucial for development and cell fate decisions. Down-regulation suggests altered developmental signaling.
    - VEGFA (Vascular Endothelial Growth Factor A): Down-regulation could imply reduced angiogenesis or altered vascular function.
    - NRL: Is a transcription factor for photoreceptor differentiation, down-regulation might indicate less focus on differentiation.
- The down-regulation of these genes suggests inhibition of certain metabolic processes, and suppression of normal neuronal development.

3. Main Functional Characteristics:

- **Modulated Neurogenesis and Synaptic Activity:** The enrichment of neurogenesis and synaptic signaling pathways is a strong functional indicator. This can reflect a state of active neuronal remodeling or adaptation.
- **Lipid Metabolism:** High expression of ACOT6 and ABCG1 suggests that these cells are actively involved in lipid metabolism, potentially for energy production or membrane remodeling.
- **Suppressed Cell-Cell Adhesion:** Downregulation of cell-cell adhesion-related genes might indicate that these cells are detaching themselves from surrounding cells.
- **Modulation of Neuronal Differentiation:** Downregulation of specific neuronal development-related genes might suggest modulation of differentiation.

4. Key Molecular Markers and Pathways:

- **Markers:** ACOT6, ABCG1, CHKB, FZD4, VEGFA, and NRL are key molecular markers.
- **Pathways:**
  - "GOBP_BEHAVIOR," "GOBP_NEUROGENESIS," and "GOBP_SYNAPTIC_SIGNALING" demonstrate the modulation of neural circuits and synaptic plasticity.
  - The enrichment of metabolic pathways supports the idea of altered metabolism within these cells.

5. Significance in a Biological or Disease Context:

These cells may be undergoing adaptation in response to external changes. These changes can be linked to diseases or physiological processes.

Overall, cluster 9 cells seem to be in a state of modulated neurogenesis and synaptic activity combined with a stress response, characterized by distinct metabolic shifts. This cell state appears to be highly adaptive and responsive to stimuli, and could be crucial for understanding human liver.

3.3 scSelector-Assisted Discovery of Cellular Subpopulation Physiological States and Biological Functions

3.3.1 scSelector Reveals Functional Heterogeneity Within Pancreatic α Cells

part1

cell state analysis result

cell state: Activated, Remodeling and Interacting State confidence: 90%

key features: • ECM Remodeling • Cellular Morphogenesis and Adhesion • Signaling

Activation • Intercellular Interactions

key pathways: • GOCC_EXTERNAL_ENCAPSULATING_STRUCTURE • GOBP_CELL_MORPHOGENESIS • GOBP_CELL_ADHESION • GOCC_COLLAGEN_CONTAINING_EXTRACELLULAR_MATRIX

Okay, I will analyze the provided single-cell RNA sequencing data to determine the biological state and functional characteristics of the selected cell group (Group 1, Cluster 0). I will focus on the gene expression patterns, pathway enrichment results, and cluster distribution information to provide a comprehensive analysis, specifically avoiding premature cell type assignments and concentrating on cell *state*.

Conclusion:

Based on the differential gene expression and pathway enrichment analysis, the selected cell group (Cluster 0) appears to be in a highly **activated, remodeling and interacting state**, exhibiting a significant extracellular matrix (ECM) organization and remodeling signature, coupled with cellular morphogenesis and adhesion programs, potentially driven by complex signaling cascades. Considering there are only 2 distinct groups, these cell states are the most representative states.

Detailed Analysis:

1. Cell State:

The cells in Cluster 0 exhibit a unique state characterized by:

- **Activation:** The high number of upregulated genes (6552) compared to downregulated (831) suggests an overall activation state, with significant transcriptional reprogramming.
- **Remodeling:** Enrichment of ECM-related pathways (GOCC_EXTERNAL_ENCAPSULATING_STRUCTURE, GOCC_COLLAGEN_CONTAINING_EXTRACELLULAR_MATRIX) points towards extensive remodeling of the extracellular environment.
- **Interacting:** Enrichment of cell adhesion pathways (GOBP_CELL_ADHESION) means that it enhances cell adhesion ability.

2. Contribution of Up-regulated and Down-regulated Genes:

- **Up-regulated Genes:** These genes drive the observed cellular state.
    - *ECM Remodeling:* Genes like *FN1* (Fibronectin 1), *COL12A1* (Collagen Type XII Alpha 1 Chain), *LAMA3* (Laminin Subunit Alpha 3), and other

collagen family members indicate significant synthesis and organization of the ECM. *ADAMTS* family genes suggests active ECM turnover. The high expression of PLAC8 suggest cell adhesion, migration, and invasion. The cell also showed JAG2, a Notch ligand known to influence cell fate decisions and tissue organization.

  - *Signaling and Activation: FCGR1A* (Fc Fragment Of IgG Receptor Ia) suggests these cells are responsive to immunoglobulin signals, possibly reflecting interactions with immune cells or antibodies. *PKN3* (Protein Kinase N3) is involved in cell motility and adhesion.
  - *Protein turnover:* Genes like *ATP6V1B2* are related to protein turnover.

- **Down-regulated Genes:** Downregulation of certain genes also contribute to the overall cellular phenotype.
  - *SMARCB1*: a tumor suppressor gene and downregulation suggests a loss of growth regulation.
  - *SEC14L5*: its downregulation suggests changes in lipid metabolism.
  - *PPIL2*: Peptidylprolyl Isomerase Like 2's downregulation likely affects protein folding and cell cycle progression.

3. Main Functional Characteristics:

- **ECM Organization and Remodeling:** This is a dominant feature, involving both synthesis and degradation of ECM components.
- **Cellular Morphogenesis and Adhesion:** The cells are actively changing shape and interacting with their surroundings and other cells.
- **Intracellular Signaling:** complex signaling cascades are active.

4. Key Molecular Markers and Pathways:

- **Key Up-regulated Markers:**
  - *PLAC8*: High Log2FC suggests a strong defining marker.
  - *JAG2*: Indicates Notch signaling activation.
  - *FCGR1A*: Suggests responsiveness to IgG signals.
  - *FN1, COL12A1:* Central to ECM organization.
- **Key Down-regulated Markers:**

- *SMARCB1, SEC14L5, PPIL2*: The downregulation suggests significant regulatory changes.

- **Key Pathways:**
  - *GOCC_EXTERNAL_ENCAPSULATING_STRUCTURE, GOCC_COLLAGEN_CONTAINING_EXTRACELLULAR_MATRIX, GOBP_CELL_MORPHOGENESIS, GOBP_CELL_ADHESION, GOBP_TISSUE_MORPHOGENESIS:* Emphasize the ECM remodeling, and cellular behavior.

5. Biological/Disease Significance:

This cellular state, marked by ECM remodeling and cellular morphogenesis, can have diverse implications:

- **Wound Healing and Tissue Repair:** These cells might be involved in repairing tissue damage.
- **Fibrosis:** Uncontrolled ECM remodeling can lead to fibrosis.
- **Tumor Microenvironment:** Cancer cells often remodel the ECM to promote tumor growth and metastasis.
- **Development:** In the context of a developing pancreas, ECM remodeling and cellular morphogenesis are essential for proper organogenesis.

6. Subgroup Analysis:

The presence of cells from Clusters 1, 3, and 11 in the comparison group suggests potential heterogeneity within the selected cell population (Cluster 0). Since Cluster 0 is selected, we can treat cluster 1,3, and 11 as subgroups of cluster 0.

- *Cluster 1-like cells (45.76%):* Given enrichment analysis shows that the gene in cluster 1 is relevant to cell morphogenesis, the "activated, remodeling and interacting state" of cluster 0 may be in the process of morphogenesis and tissue development.
- *Cluster 3-like cells (43.54%):* The presence of these cells indicate there are a large proportion of cell involved in neurogenesis and cell morphogenesis.
- *Cluster 11-like cells (10.52%):* The presence of these cells indicate there are a small proportion of cell involved in cell junction organization.

part2

cell state analysis result

cell state: Activated, adhesion-related and matrix remodeling state possibly related to wound healing and immune response confidence: 85%

key features: • Cell adhesion • Matrix remodeling • Immune modulation • Wound healing

key pathways: • GOBP_CELL_ADHESION • GOCC_EXTERNAL_ENCAPSULATING_STRUCTURE • GOBP_RESPONSE_TO_WOUNDING • GOBP_WOUND_HEALING • GOBP_BASEMENT_MEMBRANE • GOBP_REGULATION_OF_INTRACELLULAR_SIGNAL_TRANSDUCTION

Okay, I've analyzed the provided single-cell RNA sequencing data, focusing on cell state and function. My analysis avoids speculating about cell type and emphasizes the functional aspects indicated by the gene expression profiles and pathway enrichment.

Conclusion:

Based on the gene expression patterns and pathway enrichment, these cells appear to be in an **activated, adhesion-related and matrix remodeling state, possibly related to wound healing and immune response**. There is a significant downregulation of genes involved in basic cellular metabolic process. Furthermore, the subgroup analysis suggests the presence of two major populations: one highly enriched in adhesion and immune modulation, and a smaller subpopulation displaying pro-inflammatory state. This state could be observed in wound microenvironment where cellular adhesion, migration, and immune interaction are important.

Detailed Analysis:

1. **Cell State:** The up-regulation of genes involved in cell adhesion (see below), coupled with the enrichment of pathways like "GOBP_CELL_ADHESION," "GOBP_WOUND_HEALING," and "GOBP_RESPONSE_TO_WOUNDING" strongly suggests an **activated state focused on adhesion and tissue remodeling**. Downregulation of mitochondrial genes suggests decreased metabolic activity in basic cell function.

2. **Contributions of Up- and Down-regulated Genes:**

    - **Up-regulated Genes:** The highly up-regulated genes like *MGC21881*, *RANBP10*, *DGKH*, *ZFP91-CNTF*, *PAICS*, *ERCC-00085*, *ZNF599*, *GTF3C4*,

TOP3A, *RFESD*, and *CPSF3L* points to activation of matrix remodeling, regulatory transcriptional networks, and possibly a response to cellular stress or damage. *DGKH*, for example, is involved in lipid signaling and inflammation. *TOP3A* suggests DNA modification and transcriptional regulation. *RANBP10* suggests regulation of protein transport.

- **Down-regulated Genes:** The strongly down-regulated genes, including *CXXC4*, *RAPGEF2*, *PTPRH*, *TRIM34*, *TMEM194A*, *XDH*, and multiple ribosomal protein-related genes indicates a suppression of cellular metabolism, particularly oxidative phosphorylation. *CXXC4* acts as Wnt signaling inhibitor, and its downregulation might be an indicator of Wnt signaling pathway activation, which is usually observed in wound healing microenvironment. Decreased ATP production might be due to altered cell fate program.

3. **Main Functional Characteristics:**
    - **Cell Adhesion and Matrix Remodeling:** This is dominant, supported by numerous up-regulated adhesion-related genes and corresponding pathway enrichment.
    - **Immune Modulation:** The involvement of genes like *IL6*, *TNFSF11*, HLA genes, and related pathways indicates an active role in modulating the immune microenvironment.
    - **Wound Healing Response:** The enriched pathways directly related to wound healing highlight an effort to repair tissue damage, potentially by coordinating cell migration, matrix deposition, and immune cell recruitment.
    - **Suppressed cellular metabolism**: Downregulation of many genes involved in oxidative phosphorylation and ATP production, like *ATP5J2*, *UQCRH*, indicates suppressed cellular metabolism.
        - **Matrix Remodeling Related Genes:** Downregulation of matrix-related genes like *COL3A1* and *CHST11* indicates active matrix remodeling.
        - **Regulators and Transcription Factors**: Downregulation of regulator factors like *BAP1*, *ZNF454* and *MEX3D* indicated state of cell programming.
4. **Key Molecular Markers and Pathways:**

- **Key Up-regulated Markers:**
    - *DGKH*: Lipid signaling, inflammation.
    - *ZFP91-CNTF*: Transcription regulation, cell cycle control.
    - *MGC21881*: Function yet to be determined.
    - *TOP3A*: DNA repair and modification.
- **Key Down-regulated Markers:**
    - *CXXC4*: Wnt signaling inhibition.
    - *PTPRH*: Protein tyrosine phosphatase activity; signal transduction.
    - *XDH*: Purine metabolism; redox regulation.
    - *BAP1*: Tumor suppressor; epigenetic regulation
- **Key Pathways:**
    - GOBP_CELL_ADHESION
    - GOCC_EXTERNAL_ENCAPSULATING_STRUCTURE
    - GOBP_RESPONSE_TO_WOUNDING
    - GOBP_WOUND_HEALING
    - GOBP_BASEMENT_MEMBRANE
    - GOBP_REGULATION_OF_INTRACELLULAR_SIGNAL_TRANSDUCTION

5. **Significance of Cell State:** This activated, adhesion-related state is significant in biological contexts involving tissue repair, inflammation, and immune responses. It may be observed during wound healing processes, inflammation microenvironment and various tumor microenvironment.
6. **Subgroup Analysis:**
    - **Cluster 1 (81.05%):** This dominant population likely represents the core of the activated, adhesion-related cell state. High cell adhesion score. This indicates the cell are actively participating in cell-cell interaction, likely with immune cells or other stromal cells.
    - **Cluster 11 (18.63%):** A smaller subgroup that might represent a more pro-inflammatory subpopulation within the selected cells. Some pro-

inflammatory signals may be enriched in this subpopulation. Further analysis should be performed with markers to distinguish them.

  - **Cluster 8 (0.33%):** Given its tiny proportion, it is difficult to determine its characteristics. It likely a very minor subpopulation.

3.3.2 Identification of a Distinct Wound Healing-Associated α-Cell Subcluster

cell state analysis result

cell state: Neuronal signaling and ion transport specialized state, transcriptionally active
confidence: 85%

key features: • High transcriptional activity • Specialized neuronal signaling functions • Ion transport dominance • Downregulation of basal metabolism

key pathways: • DNA binding transcription factor activity • Cell-cell signaling • Synaptic signaling • Neurotransmitter transport • Monoatomic ion transport

Okay, I will analyze the provided single-cell RNA sequencing data, focusing on the biological state and functional characteristics of the cells in Cluster 10 (Group 1). I will avoid making cell type assignments and instead concentrate on the observed gene expression patterns and pathway enrichments.

Summary Conclusion:

Based on the gene expression profile and pathway enrichment, the cells in Cluster 10 appear to be in a **highly transcriptionally active state related to neuronal signaling and ion transport processes, while showing down-regulation of basal metabolic and structural processes.** This suggests a specific functional specialization related to neuronal communication.

Detailed Analysis:

1. Cell State:

- **Transcriptionally Active:** The enrichment analysis highlights pathways like "DNA binding transcription factor activity", "Cis-regulatory region sequence-specific DNA binding," and "Transcription regulator activity." The sheer number of differentially expressed genes (2743 upregulated, 1563 downregulated)

strongly suggests a dynamic and transcriptionally active cellular state.

2. Contributions of Up-regulated and Down-regulated Genes:

- **Up-regulated Genes:** The upregulated genes point towards specialized functions.
  - HMSD (highly upregulated) could be related to cell differentiation or survival, requires further context.
  - MITF: While known as a melanocyte transcription factor, it can also be involved in lysosomal biogenesis and cell survival in other contexts. Its presence suggests potential specialization or stress response.
  - KCNIP1 and GNB3: are involved in neuronal signaling, support neuron related activities
  - FOXE1 : Associated with development and transcription regulation, this could indicate ongoing cellular specification.
- **Down-regulated Genes:** The downregulated genes suggest a reduction in certain fundamental cellular processes.
  - TPI1 (highly downregulated): This is a key glycolytic enzyme. Its downregulation suggests a reduced reliance on glycolysis, which could imply a shift towards alternative metabolic pathways.
  - CTNS: Transports cystine across lysosomal membranes. It's downregulation may impair lysosomal functions.
  - DOT1L, SMARCE1: are linked to epigenetic regulation, their reduction may contribute to changes in gene expression.
  - CIC: CIC acts as a transcription repressor.

3. Main Functional Characteristics:

- **Neuronal Signaling Specialization:** The enrichment of "Cell-cell signaling," "Synaptic signaling," and "Neurotransmitter transport" pathways indicate a strong functional specialization related to neuronal communication. This is further supported by the upregulation of genes like *GNB3* and *KCNIP1*.
- **Ion Transport Dominance:** Pathways like "Monoatomic ion transport", "Sodium ion transmembrane transporter activity", and "Metal ion transmembrane transporter activity" are prominent. This emphasizes the importance of ion homeostasis and signaling in these cells.

4. Key Molecular Markers and Pathways:

- **Key Up-regulated Markers:**
    - HMSD: requires further investigation into function in the liver
    - MITF: While known as a melanocyte transcription factor, it can also be involved in lysosomal biogenesis and cell survival in other contexts. Its presence suggests potential specialization or stress response.
    - KCNIP1 : important for neuronal signaling, may relate to specific signal receiving.
    - GNB3: G protein subunit.
    - FOXE1: Transcription factor involved in development.
- **Key Down-regulated Markers:**
    - TPI1: Glycolysis.
    - CTNS: lysosomal function.
    - DOT1L, SMARCE1: Epigenetic regulation.
    - CIC: CIC acts as a transcription repressor.
- **Key Pathways:**
    - "DNA binding transcription factor activity", "Cis-regulatory region sequence-specific DNA binding": High transcriptional activity.
    - "Cell-cell signaling", "Synaptic signaling", "Neurotransmitter transport": Specialized neuronal communication functions.
    - "Monoatomic ion transport", "Sodium ion transmembrane transporter activity": Ion homeostasis and signaling.

5. Significance of Cell State:

- **Biological/Disease Context:** In the context of the liver, the finding of cells specialized for neuronal signaling and ion transport is somewhat unusual. This cell state could represent:
    - **Aberrant Cellular Differentiation:** It could be related to unusual differentiation event within the liver.
    - **Response to Specific Stimuli:** Specific stimuli like drug exposure or inflammation could cause hepatocytes to transition to this state.

- **Interaction with other cells:** specialized neuronal signaling and ion transport facilitates cell interaction, which can be critical during liver regeneration.

3.5 scSelector-Assisted Discovery of Biological Functions in Cells Discarded During Pre-processing

cell state analysis result

cell state: Activated state with strong cell adhesion, extracellular matrix remodeling, and immune interactions confidence: 90%

key features: • Cell adhesion • Extracellular matrix remodeling • Immune interactions • Activation of transcription factors

key pathways: • Cell adhesion • External encapsulating structure organization • DNA binding transcription factor activity • Phosphorylation • Skeletal system development

Okay, I will analyze the provided single-cell RNA sequencing data and provide a comprehensive assessment of the cell state and function based on the differential gene expression and pathway enrichment results. My analysis will focus on the functional characteristics of the cells, rather than cell type identity.

Summary Conclusion:

Based on the gene expression and pathway enrichment results, these cells appear to be in an **activated state with strong cell adhesion, extracellular matrix remodeling, and potential interactions with the immune system**. The up-regulation of genes involved in cell adhesion, extracellular matrix organization, and immune-related processes suggests an active role in tissue remodeling, cell-cell communication, and potentially inflammatory responses. Down-regulation of genes related to cell cycle and TGF-beta signaling may indicate a shift away from proliferation and certain developmental processes.

Detailed Analysis:

1. Cell State:

The cells are likely in an activated state. This is supported by several lines of evidence:

- **Up-regulation of genes involved in cell adhesion (e.g., *ITGB1*, *CDH6*)**: This

suggests the cells are actively interacting with their surrounding environment and other cells.

- **Up-regulation of genes involved in extracellular matrix organization (e.g., *COL1A1*, *LAMA2*)**: This indicates an active role in tissue remodeling and structural support.
- **Up-regulation of genes involved in signaling and transcription (e.g., *PAX3*, several zinc finger proteins)**: Activation of signaling pathways and transcription factors is a hallmark of cellular activation.
- **Differential gene regulation indicates a switch in cellular programs**: With so many up-regulated genes against a much smaller set of down-regulated genes, it strongly implies a shift to an active state.

2. Contributions of Up-regulated and Down-regulated Genes:

- **Up-regulated Genes:** The significantly enriched "Cell Adhesion" and "External Encapsulating Structure Organization" pathways due to the up-regulated genes indicate these cells are strongly involved in processes like cell-cell and cell-matrix interactions. Genes like *ITGB1* (Integrin beta 1) are crucial for cell adhesion and migration. Up-regulation of *HLA-DOA* and other HLA genes suggests interactions with the immune system and antigen presentation. Genes like *PAX3* suggests activation of specific developmental transcription programs which also might be related to cell survival and adaptation. The up-regulation of *ALDOA*, a key enzyme in glycolysis, hints at increased energy demands, potentially supporting the activated state. Also of note is *CSAD*, a gene coding for cysteine sulfinic acid decarboxylase, involved in taurine biosynthesis, which itself has roles in antioxidation, membrane stabilization and calcium modulation.
- **Down-regulated Genes:** The down-regulation of genes such as *QPCTL*, *LMNB1* and *CEP55*, which are involved in protein glutaminyl cyclase activity, nuclear structure and cell division respectively, suggests a possible shift away from proliferation and specific protein modifications. Down-regulation of *TGFBR2* suggests a reduced responsiveness to TGF-beta signaling, which could influence cell growth, differentiation, and immune regulation. Downregulation of *PRR23C* suggests these cells are in a different proliferation state compared to others.

3. Main Functional Characteristics:

- **Cell Adhesion and Extracellular Matrix Remodeling:** This is a dominant characteristic, indicated by the strong enrichment of related pathways and the up-regulation of genes like collagens, integrins, and matrix metalloproteinases.

- **Immune Interactions:** The up-regulation of HLA genes and other immune-related genes suggests these cells may be involved in immune surveillance or modulation.
- **Metabolic Activity:** The up-regulation of glycolytic enzymes suggests a higher energy demand, consistent with an activated state.
- **Transcription Factor Activation:** The enrichment of pathways related to DNA binding transcription factor activity suggests active regulation of gene expression, which is characteristic of a cell responding to stimuli.

4. Key Molecular Markers and Pathways:

- **Key Up-regulated Markers:**
  - *ITGB1:* Integrin beta 1, a critical mediator of cell adhesion and signaling.
  - *HLA-DOA:* MHC Class II molecule, involved in antigen presentation and immune interactions.
  - *ALDOA:* Fructose-bisphosphate aldolase A, involved in glycolysis.
  - *PAX3:* Involved in cell proliferation and survival.
  - *CSAD:* Involved in taurine biosynthesis, important for cytoprotection.
- **Key Down-regulated Markers:**
  - *TGFBR2:* TGF-beta receptor II, involved in growth, differentiation, and immune regulation.
  - *CEP55:* Centrosomal protein 55, crucial for cell division.
  - *QPCTL:* Glutaminyl-peptide cyclotransferase, involved in protein modification.
  - *LMNB1:* Lamin B1, maintaining nuclear structure
  - *PRR23C:* Proline Rich 23C, related to cell proliferation
- **Key Pathways:**
  - Cell Adhesion
  - External Encapsulating Structure Organization
  - DNA Binding Transcription Factor Activity
  - Phosphorylation

- Collagen Fibril Organization

5. Significance in a Biological or Disease Context:

This cell state could be significant in several contexts:

- **Tissue Repair:** The activated state with increased cell adhesion and extracellular matrix remodeling suggests a role in tissue repair processes.
- **Inflammation:** The immune interactions and up-regulation of inflammatory mediators could indicate a role in inflammation.
- **Cancer:** In a tumor microenvironment, this cell state might reflect tumor-stromal interactions, matrix remodeling to promote tumor progression, or an immune response against the tumor.
- **Fibrosis:** In fibrotic diseases, this state could contribute to excessive matrix deposition and tissue scarring.

In summary, the cells exhibit an activated state characterized by cell adhesion, extracellular matrix remodeling, immune interactions, and transcriptional regulation. This state likely plays a role in tissue repair, inflammation, or disease processes such as cancer and fibrosis. Understanding the specific triggers and downstream effects of this state could provide insights into potential therapeutic targets.
