# Supplementary material for: scSelector: A Flexible Single-Cell Data Analysis Assistant for Biomedical Researchers
Source: Genes (Basel). 2025 Dec 19;17(1):2. doi: 10.3390/genes17010002 (PMC12841117; doi:10.3390/genes17010002)
Supplement: Supplementary file 1 [file genes-17-00002-s001.zip › Supplementary File S5.pdf]

For software:

Project name: scSelector.

Project home page: https://github.com/miao1332211/scSelector (accessed on 11 December 2025).

Archived version:

Operating system: Windows, macOS, and Linux.

Programming language: Python.

Other requirements: Anaconda (or Miniconda) for environment management. An API key from DeepSeek or Gemini is required for the AI-powered analysis functions.

License: MIT License.

Any restrictions to use by non-academics: None.
