# Supplementary figures and images for: scSelector: A Flexible Single-Cell Data Analysis Assistant for Biomedical Researchers
Source: Genes (Basel). 2025 Dec 19;17(1):2. doi: 10.3390/genes17010002 (PMC12841117; doi:10.3390/genes17010002)

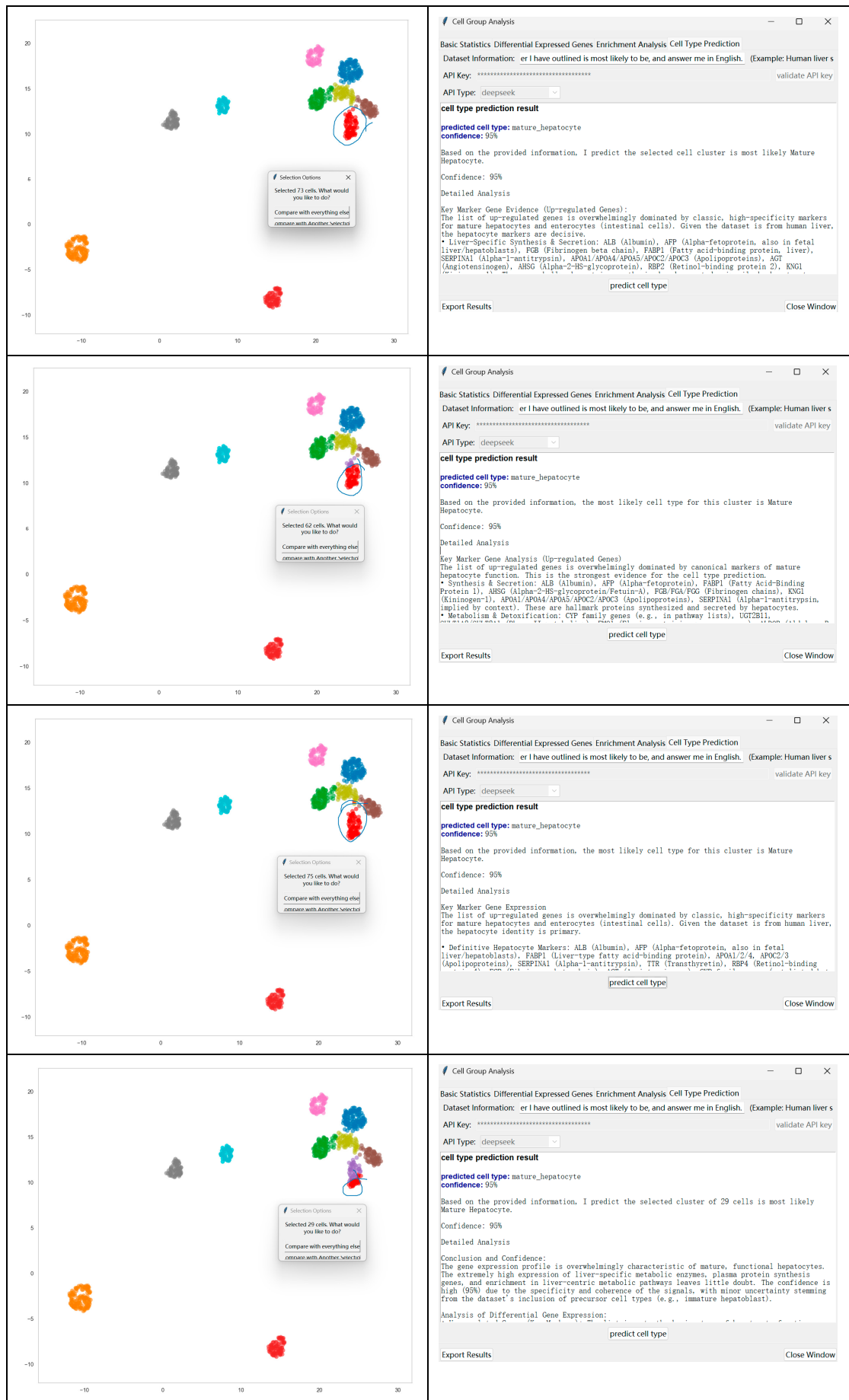

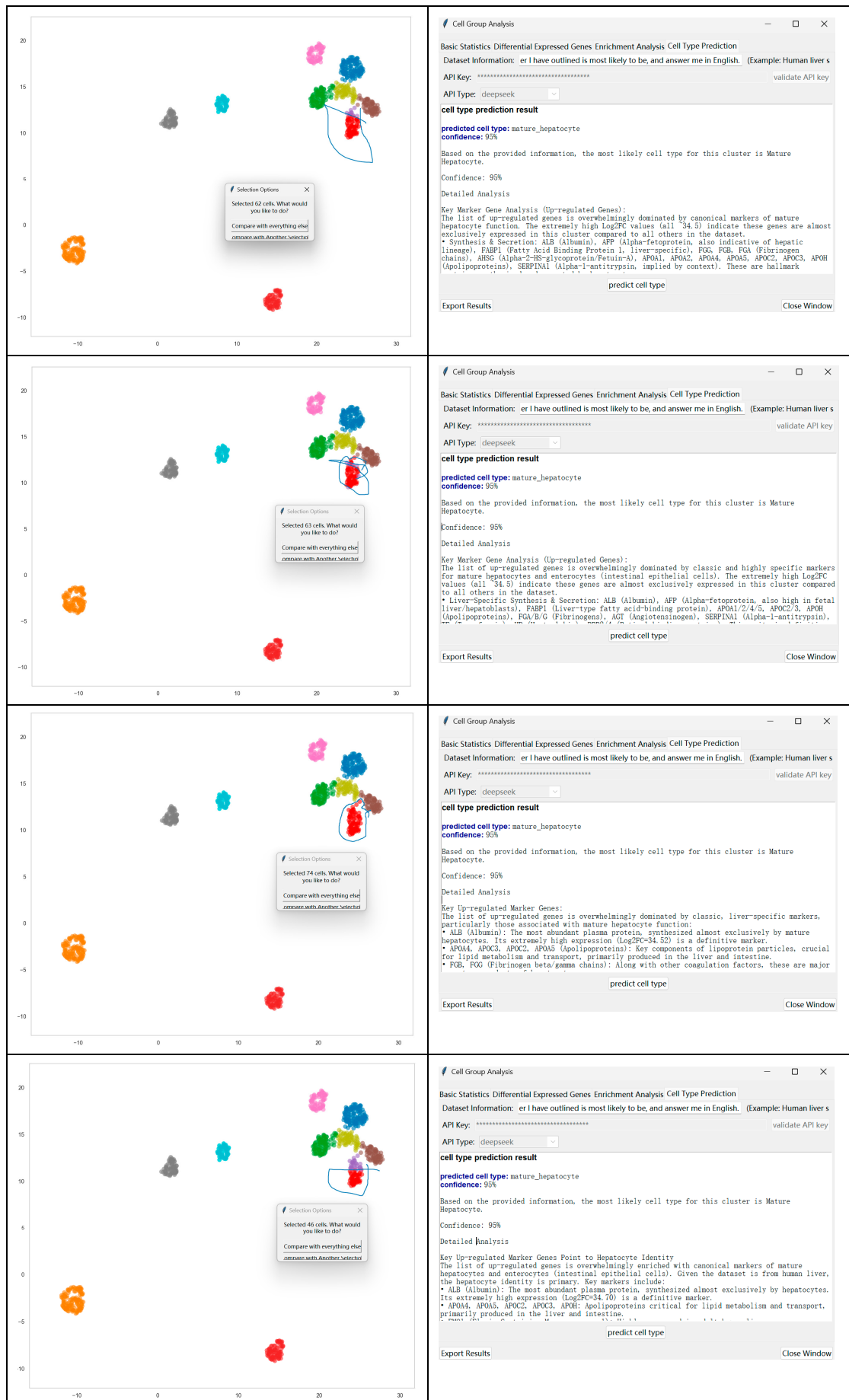

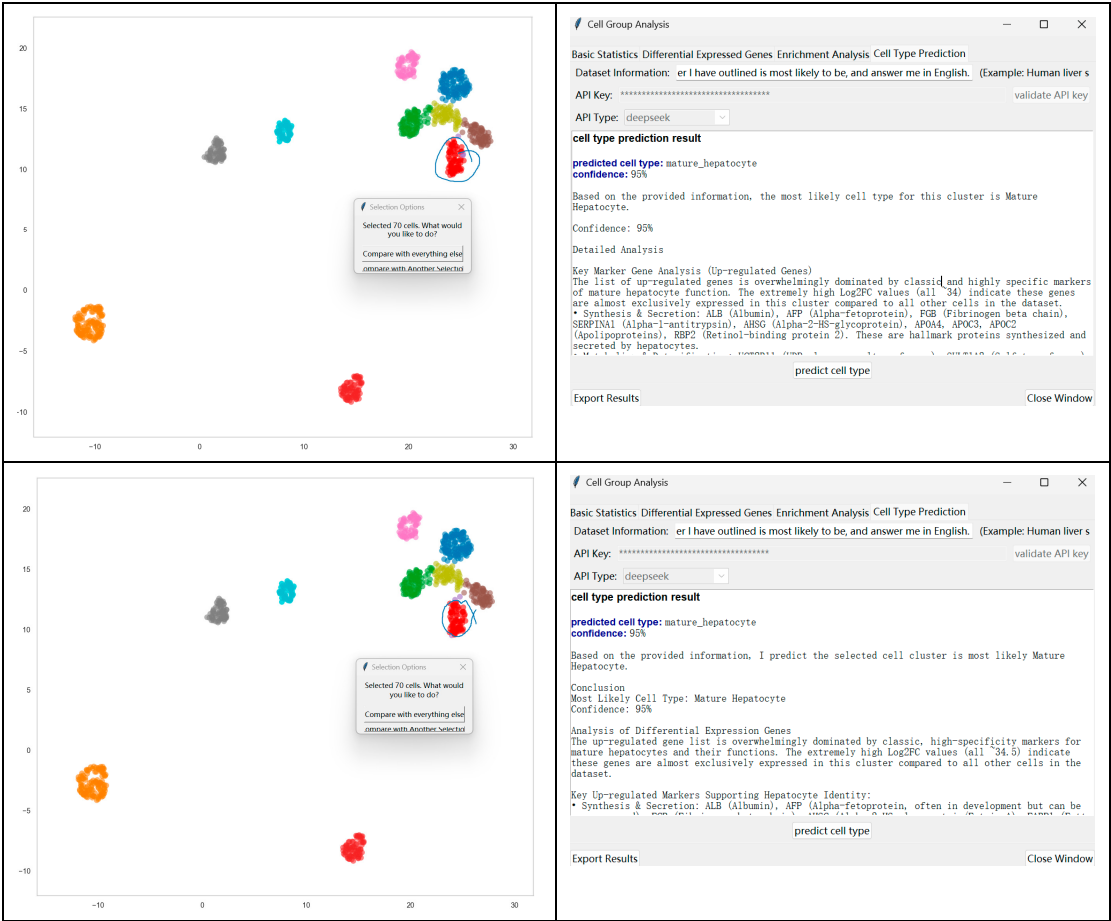

Supplement: Supplementary file 1 [file genes-17-00002-s001.zip › Supplementary File S4.pdf]
